# Comparative Life Cycle Assessment of a Novel Al-Ion and a Li-Ion Battery for Stationary Applications

**DOI:** 10.3390/ma12193270

**Published:** 2019-10-08

**Authors:** Mario Amin Salgado Delgado, Lorenzo Usai, Qiaoyan Pan, Anders Hammer Strømman

**Affiliations:** 1 Industrial Ecology Program, Norwegian University of Science and Technology, E1-Høgskoleringen 5, 7491 Trondheim, Norway; lorenzo.usai@ntnu.no (L.U.); anders.hammer.stromman@ntnu.no (A.H.S.); 2 ACCUREC Recycling GmbH, Bataverstraße 21, DE-47809 Krefeld, Germany; qiaoyan.pan@accurec.de

**Keywords:** industrial ecology, life cycle assessment, aluminium-ion, lithium-ion, cradle-to-grave, stationary battery, decentralized energy systems

## Abstract

The foreseen high penetration of fluctuant renewable energy sources, such as wind and solar, will cause an increased need for batteries to store the energy produced and not instantaneously consumed. Due to the high production cost and significant environmental impacts associated with the production of lithium-ion nickel-manganese-cobalt (Li-ion NMC) batteries, several chemistries are proposed as a potential substitute. This study aims to identify and compare the lifecycle environmental impacts springing from a novel Al-ion battery, with the current state-of-the-art chemistry, i.e., Li-ion NMC. The global warming potential (GWP) indicator was selected to express the results due to its relevance to society, policy and to facilitate the comparison of our results with other research. The cradle-to-grave process-based assessment uses two functional units: (1) per-cell manufactured and (2) per-Wh of storage capacity. The results identified the battery’s production as the highest carbon intensity phase, being the energy usage the main contributor to GWP. In general, the materials and process involved in the manufacturing and recycling of the novel battery achieve a lower environmental impact in comparison to the Li-ion technology. However, due to the Al-ion’s low energy density, a higher amount of materials are needed to deliver equivalent performance than a Li-ion.

## 1. Introduction

To mitigate climate change, modern society must face dramatic shifts in its socioeconomic metabolism, being an energy transition at the core of this change. As an example, the International Energy Agency forecasts that in 2040 between 8000 and 14,000 TWh of final electricity demand could be provided by wind and solar photovoltaic (PV) sources [1]. Since wind and solar energy are intermittent, electrochemical energy storage is emerging as an option to develop decentralized electricity generation systems [2]. 

Currently, a wide spectrum of chemistries can be potentially used in stationary applications, offering consequently, different environmental impacts associated with their intrinsic features and lifecycle phases [3]. This is the case of sodium, magnesium or calcium-ion chemistries which are promising technologies due to their appalling abundancy that is essential for large-scale penetration of stationary batteries [4,5,6]. Moreover, Li-ion technology remains the preferred choice due to its high energy density, decent cycle life and the possibility of operating safely at high voltages [7,8]. However, supply risks, high production costs and considerable environmental impacts represent a major concern for stakeholders and policymakers [9]. 

The study presented here is part of the European ALION project, which aims to develop a rechargeable high specific energy Aluminium-ion (Al-ion) battery for decentralized electricity applications. In the context of sustainability, this work had the objective of performing a full and comprehensive life cycle assessment (LCA) to frame potential benefits and pitfalls of a novel Al-ion battery in comparison with the existing preferred Li-ion chemistry. In line with this goal, Section 2 briefly summarizes the most relevant findings and characteristics of previous environmental assessments on batteries for stationary applications and enhances the importance of our work as the first LCA on Al-ion batteries. Section 3 explains the method, the system modelled, and the inventory compiled. The results are presented and discussed in Section 4 and finally, in Section 5 the conclusions and key recommendations are presented to ensure the sustainable development of the novel battery.

## 2. State of Knowledge

In recent years, the environmental impacts associated with the production, use and disposal of batteries have been broadly assessed [10,11,12,13,14,15,16,17,18,19]. However, the research has mainly focused on mobility applications [20]. After a deep literature review, this study found that only a few studies estimate the environmental performance of batteries in stationary systems and that no research has been performed to assess the environmental performance of an Al-ion battery. Hence, in this section, the state of knowledge of LCA studies of batteries focused on the aforementioned application is briefly presented.

One of the first cradle-to-grave studies about stationary storage systems was performed by Longo et al. [21] who estimated the potential impacts of a sodium/nickel chloride battery in a photovoltaic system. The authors used as a functional unit one battery of the defined technology. Their results showed that the manufacturing phase is the greatest environmental impact contributor, accounting for more than 60% of the total impacts. The authors concluded that such impact could decrease if the battery’s manufacturing process becomes more energy-efficient and if the energy inputs in this phase rely on renewable sources. Later, Hiremath et al. [3] estimated the cumulative energy demand and global warming potential (GWP) of four stationary battery technologies (Li-ion, lead-acid, sodium-sulphur and vanadium-redox-flow), in seven different applications and modelling three different types of power sources (German distribution grid, solar, solar-wind-mix). The study was performed from a comparative life cycle assessment perspective and the functional unit set was one megawatt-hour of electricity delivery. Due to lack of data, the authors decided to ignore the end-of-life (EoL) phase. For all the analysed batteries, the results highlighted that when the power source is other than renewable, the use phase dominates the environmental impacts. In addition, the authors emphasized the need for deploying batteries with higher Coulombic efficiency, similar to Li-ion’s technology. Recently, Vandepaer et al. [22] assessed the environmental performance of a novel lithium metal polymer (LMP) battery and compared it with Li-ion battery through the LCA framework. The cradle-to-grave assessment encompasses 15 impact categories and models two scenarios: One where the storage capacity is 6 MWh in a centralized application; and a second with 75 kWh capacity for a distributed grid configuration. Similar to the findings of Longo et al. [21], it was found that the manufacturing phase has the highest carbon intensity. In terms of GWP and ozone depletion, the Li-ion battery registered higher contributions. Finally, Vandepaer et al. concluded that when a battery application is in centralized system configurations, the environmental impact is relatively smaller than in distributed systems with more but smaller (energy content-wise) units. Simultaneously, Peters and Weil [23] exanimated for the first time the potential environmental impact of an aqueous hybrid ion battery (AHIB) under a detailed prospective LCA framework. That study compared the AHIB with two different chemistries (lithium-ion and sodium-ion) in two scenarios: (1) a hypothetical residential PV application and (2) a hypothetical island microgrid composed by PV and a diesel-based power generation system. The comparison was done in a mass and energy capacity basis (i.e., kg and kWh). Peters and Weil found that the environmental performance of the AHIB is poorer for GWP and ozone depletion due to higher internal inefficiencies, which are important factors when the energy source is from fossil fuels. Finally, the authors of that study enhanced the importance of the functional unit applied. For example, on a mass basis, it was proven that AHIB has a lower impact in four out of six of the impact categories covered. 

To date, the research on the environmental impact of batteries in stationary applications has not been deployed on a large scale. Furthermore, the environmental characterization of Al-ion technology has never been performed. This study addresses this research gap by performing a comparative LCA based on primary data from both laboratory and commercial scales of a novel Al-ion chemistry for stationary applications.

## 3. Methods and Data

LCA is an environmental accounting and management approach that takes into account, from a holistic perspective, the relevant flows associated with the life cycle stages of a good or service and translates them into relevant potential environmental impacts [24,25,26]. This study performed a process-based LCA to estimate the environmental characterization of the novel Al-ion battery. To assess the environmental performance of the novel technology, Li-ion chemistry was chosen as a reference. The assessment was carried out from a cradle-to-grave perspective using the ReCiPe v1.1 framework [27] and partners from the ALION consortium contributed with valuable primary data about raw materials usage, manufacturing steps, the use phase and recycling processes of the novel battery. For the reference chemistry, in-house primary data was used. The associated upstream processes and emissions were backed with ecoinvent 3.2 database [28]. This study presents the results in GWP terms due to their relevance to society, policy and to facilitate the comparison of the results with other research. However, numerical results for other seven impact categories are available in the Supplementary Materials (SM), Section 2. 

### 3.1. Functional Unit

The functional unit (FU) represents the reference used to measure the environmental performance of the system under assessment and in combination with the choice of the impact categories. It is a fundamental step of the goal and scope definition on an LCA. Furthermore, the functional unit must ensure the comparability of the results with other similar systems [13]. This study chose to express the results in two FUs. First, using a per-Wh of storage capacity basis, in order to capture the relative performance of the technology as was done by Majeau-Bettez, Hawkins, and Strømman [13]; Ellingsen et al. [10]; Hiremath et al. [3]; Kim et al. [12] and Peters and Weil [23]. Second, a per-cell basis was chosen, showing the potential benefits of the novel chemistry throughout the entire life cycle of one cell. This type of functional unit has been recorded in similar studies such as Bauer [17]; Ellingsen et al. [10]; Peters and Weil [24] and Zackrisson, Avellán, and Orlenius [16]. It should be mentioned that the novel cell is in a low technology readiness level (TRL) and its energy performance is still well below compared to the state-of-the-art Li-ion chemistry. Therefore, it is considered that the use of both FUs brings a fair and impartial comparison of the environmental performance of both chemistries from two different perspectives.

### 3.2. Battery Modelling and Inventory Compilation

The inventory compilation was time and data-intensive. Each lifecycle phase assessed required assumptions because the novel chemistry was produced on a laboratory scale and further compared with an already established technology. Therefore, using data from Ellingsen et al. [10] and following the guidelines provided by Piccinno and colleagues [29], it was possible to estimate the likely energy intensity and to scale up the production of small quantities of the chemicals used for the novel cell production. Thus, using primary data provided by ALION consortium members, the novel technology could be modelled. Specifically, for the production phase, ALION partners provided the bill of materials for the Al-ion 18650-cell manufacturing and data on the production pathway of the electrolyte. Moreover, the production of the ionic liquid used as electrolyte required an extensive literature review in order to spot the production pathway that could be used for its conversion into a life cycle inventory. Regarding the use phase, a scenario was assumed where the cell was coupled to a PV system. This consideration is in alignment with the research performed by our partner, the University of Southampton, who had the task of estimating the performance of the Al-ion chemistry. Finally, for the EoL ACCUREC designed a novel recycling process to treat spent Al-ion batteries. It should further be mentioned that to test the recycling of Al-ion batteries, ACCUREC performed experiments on a lab-scale. Therefore, the data used for the LCA comparison was scaled-up to an industrial process based on ACCUREC’s own expertise. All the lifecycle phases modelled consider the electricity required for heating and by the machinery used (modelled as a mass of steel). Furthermore, it considers the chemicals and materials consumed. Thus, the novel battery modelled for this study has a total weight of 29.3 g, specific energy of 9 Wh/kg and can last up to 5000 cycles at a depth of discharge of 80% across the entire lifetime of the battery. The nominal operating voltage is of 2V and has a nominal capacity of 300 mAh. Following, the main assumptions undertaken for the three life cycle phases assessed are presented. The detailed first- and second-tier inventories together with more specific assumptions are available in the SM, Section 1 from Tables S1–S11.

#### 3.2.1. Cell Manufacturing

**Figure 1:**
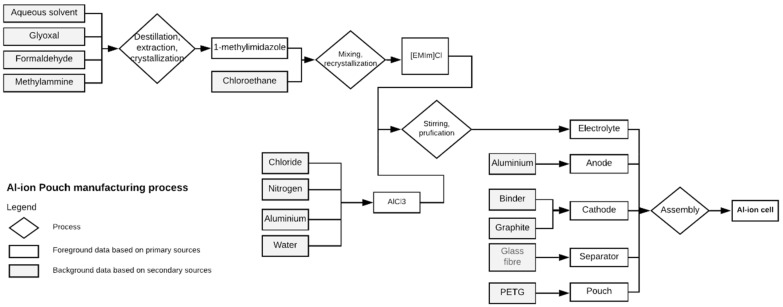
Production process of an Al-ion 18650 battery. The rhombuses represent the unit operations. The grey boxes illustrate the background datasets which are based on secondary data and the white boxes show the foreground datasets which are compiled from primary sources.

The production phase covers the five main elements that make up a 18650-cell type. These are an anode, cathode, cell canister, separator and electrolyte. Since most of the cells nowadays are produced in East Asia, the South Korean electricity mix was assumed as an energy input to the cell production phase [10]. Regarding the energy intensity, the same inputs used to model the Li-ion cell’s production was used as a reference. However, there is a noticeable difference between the manufacturing of the electrodes for the two battery technologies. This is, the production of a Li-ion battery requires the preparation and the coating of the slurry onto the current collectors which is an energy-intensive process. According to manufacturers, this stage can contribute to approximately 27% of the total energy consumption in the assembly phase. Therefore, since the novel chemistry does not use this process, a corresponding reduction was implemented into the compiled inventories. Section 1 of the SM provides a more detailed description of the manufacturing process (Figure S2). Below, Figure 1 schematizes the main processes and elements comprised in the production of the Al-ion battery. 

**Figure 2:**
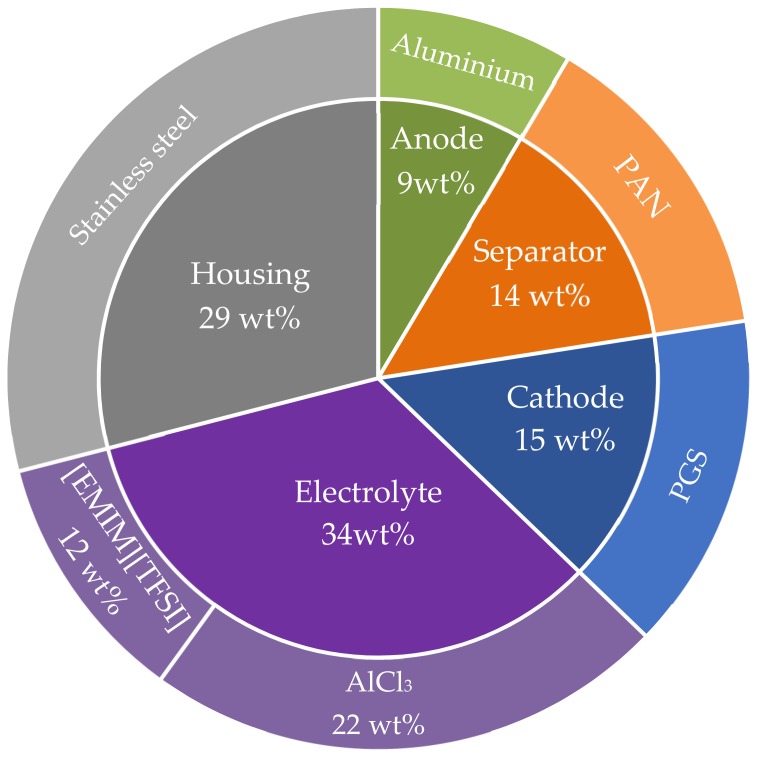
Material composition of the Al-ion 18650 battery. Weight-wise, the electrolyte is the main component accounting for the 34 wt % of the cell’s weigh. The housing shares 29 wt %, the cathode 15 wt %, the separator 14 wt % and the anode 9 wt %.3.2.2. Use Phase.

By design, the anode is the lightest component of the Al-ion cell, contributing to 9wt% of its total weight. This electrode is made of pure aluminium 3000 series. This means that its aluminium content is at least 97.8% [30]. The cathode represents 15wt% of the total cell’s weight and was modelled as pyrolytic graphite and a small amount (4wt%) of binders. For the separator, ALION partners carried out several tests to find the optimal material to act as a separator. From the different materials tested, the polyacrylonitrile was chosen as the material that meets most of the requirements with good mechanical properties. This component was modelled as 95% of acrylonitrile and 5% of methyl methacrylate and shares 14wt% of the cell’s weight. The corresponded energy consumption for its preparation and subsequent electrospinning were also considered [31]. The cell canister accounts for 29wt% of the cell’s weight, and it is made of chromium steel, like most of the 18650-cells currently on the market. Finally, the electrolyte is the heaviest component, accounting for 34wt% of the cell’s weight. Throughout the project, various ionic liquids were investigated for their potential application as an electrolyte. Among those, [EMIM][TFSI]:AlCl_3_ was identified as the most promising in regards to the cycle life of the battery and the reversibility of aluminium intercalation chemistry [32]. The electrolyte used is a mixture of EMIM[TFSI] and aluminium chloride (AlCl3). To model AlCl_3_ production, background data was used from the ecoinvent 3.5 database and for the production of EMIM[TFSI], primary data provided by ALION partners was combined with data from previous publications [33,34,35]. Furthermore, the fragmentary data required different assumptions. For example, trifluoromethanesulfonic acid - triflic acid – (TFSA) is considered as a proxy instead of TFSI. Furthermore, chloride was assumed as the halogen used to stimulate the anion exchange between EMI and TFSI. Lastly, where minor data was missing throughout the synthesis of the electrolyte, this study opted to utilize generic processes contained in ecoinvent 3.5 database. Figure 2 and Figure S1 show the material composition for Al-ion chemistry.

For this phase, we account the potential environmental impacts stemming from the extra electrical energy needed to cover charge and discharge losses [16]. Moreover, the Coulombic efficiency of the novel chemistry strongly depends on the charge/discharge rate applied. According to Holland et al. [36], the Coulombic efficiency of Al-ion technology can oscillate between 85% to 100%. This study decided to base the assumptions on the research made by the University of Southampton. Furthermore, Lin et al. [37] pointed out that the Al-ion cell has a Coulombic efficiency of 98% and reaches 7500 cycles. Nonetheless, the authors mentioned that such efficiency is theoretical. For this reason and due to the slight differences in the chemical composition between chemistries, a conservative value of 95% Coulombic efficiency and a cyclability of 5000 was used. Regarding the reference technology, a 95% Coulombic efficiency and a cyclability of 3000 [10] was used. Finally, to calculate the impacts related to the use phase, this study assumed that the cells are used in a stationary application where the energy supplied is produced by a PV installation with 3 kW as peak capacity. 

#### 3.2.2. End-of-Life Phase

**Figure 3:**
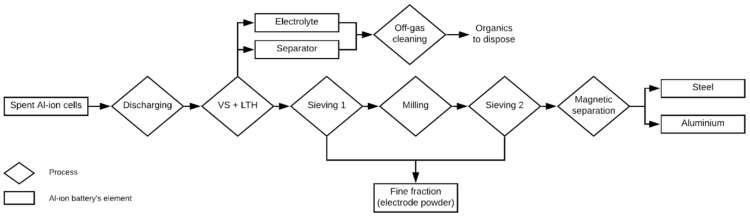
Flowchart of the novel Al-ion recycling process. The rhombuses represent the unit operations and the boxes represent the materials separated.

The last life cycle of a cradle-to-grave study is the EoL treatment of the product. For this phase, ACCUREC designed a novel process to treat spent Al-ion batteries. Due to the controversy regarding the allocation of impacts from the recycling process, the environmental profile obtained only covers the energy, materials and machinery needed for the materials’ recovery. Hence, the eventual disposal or reuse of the materials were not included within the system boundaries of this study. Figure 3 illustrates the recycling process along with its main phases. In short, the process consists of three main operations plus one cleaning system: discharging, vacuum shredding; the low-temperature heating process (VS+LTH); the mechanical separation; and an off-gas cleaning system. The four recycling stages modelled for this study are explained below.

To minimize the risk in transportation, storage and subsequent shredding treatment, the first step within the recycling process is to ensure the full discharging of the batteries. In this stage, the cell units are immersed in an electrically conductive solution (e.g., brine) for two weeks. Following this, the spent batteries enter the VS + LTH process. The shredding process runs under vacuum conditions to avoid air emissions from the electrolyte and to minimize the risk of fire. Afterwards, an LTH process, operating at 100 °C–400 °C, takes place in order to separate the electrolyte and separator. The off-gas cleaning system is an auxiliary process and primarily directed at capturing the emissions generated during the heat treatment. The system is equipped with an adsorbent to reduce the air emissions of particulate matter, odours and volatile organic compounds. When the adsorbent is saturated, it is sent to a proper facility to be reactivated or be used as secondary fuel. 

The next stage is to separate the rest of the materials into individual fractions. In this step, a mechanical separation combined unit separates the coarse fraction via a first sieving process. To increase the separation efficiency, these materials are milled and sieved again before using a magnet to attract the magnetically susceptible material, i.e., the cell canister. In the two sieving processes, it is expected that the fine fraction recovered is composed of the cathode material, i.e., pyrolytic graphite (PG). Due to the presence of impurities, the PG is planned to have a second life as a reducing agent in pyrometallurgy processes. Finally, the aluminium and the steel are sent to smelters to produce secondary metals. The EoL process is explained more profoundly in SM Section 1.1.2 and Figure S3.

### 3.3. Reference Technology

Among the large variety of chemistries available for the Li-ion technology, the (Ni0.45Co0.45Mn0.1)O_2_, or NMC, chemistry was selected because of its wide use. In addition, NTNU counts with a unique and high-resolution primary data of the production phase of this chemistry [11,38,39]. Although the Li-ion cell’s inventory is from a traction battery, it is considered a suitable proxy to represent a competitive technology in stationary applications. In particular, the idea of using traction batteries in their second life as part of stationary energy storage systems [40,41,42] has attracted attention. The material composition of the reference cell is steel for the cell canister, propylene for the separator, a mix of lithium hexafluorophosphate (LiPF6) and organic solvents as the electrolyte, coated aluminium and NMC for the cathode and coated copper for the anode. Both electrodes were modelled with their corresponded binders’ mixtures. Thus, the reference battery modelled has a total weight of 43 g, which is 14.1 g heavier than the novel technology. This is attributed to the higher amount of materials used in the anode and cathode. The specific energy is assumed to be 125 Wh/kg with a lifetime of 5000 cycles and the nominal capacity 1.5 Ah [37,38].

To model the lifecycle of the Li-ion chemistry, the manufacturing phase was assumed to require 33.4 kWh per kg of batteries produced [37] and that their assembly occurred in South Korea [10]. For the use phase, the charge/discharge efficiency of 95% was considered and that it operated in the same PV system conditions assumed for the novel battery. The EoL stage was modelled using primary data provided by our partner, ACCUREC, and was based on a generic recycling process currently in use to treat spent Li-ion batteries. Similarly, to the Al-ion recycling phase model, for the Li-ion, only the energy and materials requirements by the process were covered, and the potential environmental benefits were left out of the system boundaries of the second life of the recycled materials. Section 1.2 of the SM provide a deeper description of the reference battery (Figure S4), its manufacturing and EoL processes (Figures S5 and S6), and breakdowns the first two levels of the inventory compiled to simulate its environmental performance (Tables S13–S24)

## 4. Results and Discussion

### 4.1. Cradle-to-Gate

**Figure 4:**
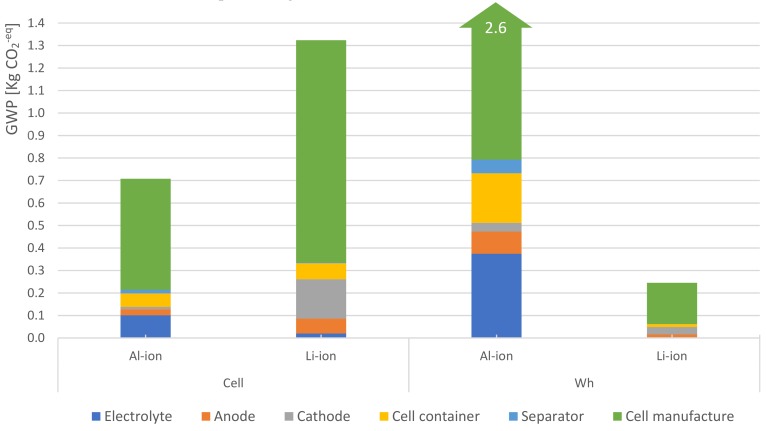
Breakdown of 18650-cell’s material composition and their absolute contribution to the global warming potential (GWP). Regardless of the functional unit or chemistry, the manufacturing process is the largest contributor to the GWP. On a per-cell basis, the Al-ion cell has a superior performance mainly because of the lack of copper in the anode. Its total contribution is about 0.7 kg of CO_2_^−eq^, while the Li-ion battery has a total contribution of 1.3 kg of CO_2_^−eq^. However, when the functional unit is based on energy content, the picture changes due to the low energy density of the Al-ion battery. Thus, in a per-watt-hour stored capacity basis, the Al-ion cell potentially has a footprint of 2.6 kg of CO_2_^−eq^, while the Li-ion battery barely contributes, with 0.25 kg of CO_2_^−eq^.

Figure 4 presents the potential impacts, in GWP terms, due to the production of Al-ion and Li-ion chemistries. The figure breakdowns the cells’ material composition and their absolute contribution to the GWP indicator for both functional units. On a per-cell basis, a clear advantage is observed for the Al-ion battery over the Li-ion. 

The main benefits can be attributed to three factors: (1) The absence of copper and NMC paste in the Al-ion battery and the use of lower carbon-intensive materials such as graphite; (2) fewer material requirements in terms of weight; (3) the electrodes of the Al-ion cells are not coated. These processes typically occur in dry room conditions, such as coating, calendering and stacking which are avoided and result in a reduction of 30% of the energy intensity. Moreover, a per-energy content approach shows that due to the low energy density of the Al-ion cell, its environmental performance is substantially poorer. In this case, the Al-ion battery has an impact 12 times larger than the reference cell. Hence, this shows that in an energy content basis, the impacts are inversely proportional to the energy density of the battery. This is under an energy content approach: The higher the energy density of the cell, the lower the environmental impacts per energy unit. 

In general, the production phase model demonstrates that, regardless of the technology or the functional unit, the assembly process (displayed in green) is the greatest contributor to greenhouse gasses (GHG) emissions due to the high carbon-intensive electricity mix used in the process. The results for the other seven impact categories can be found in the SM, Section 2.

For the use phase, our model estimates that the equivalent carbon emissions caused by the PV system and the internal inefficiencies of the novel Al-ion battery are approximately 0.008 kg of CO_2_^−eq^ while for the Li-ion, it is estimated to be 0.098 kg of CO_2_^−eq^. The potential impacts of the Li-ion’s use phase are more significant due to the substantial difference in a lifetime and because of its greater energy density which entails greater losses. In general, the use of a PV system has high relevance to keep the use phase with a low carbon footprint. As a demonstration, Hiremath et al. [3] found that the use stage of batteries can dominate their life cycle impacts when other than renewable energy sources are used. 

It should be considered that these results are in function of the modelling method chosen. Indeed, for this life cycle stage, only the cycling efficiency losses were used which due to the low energy content of the Al-ion cell, it led to a lower carbon footprint. Thus, it is expected that in the future, as the Al-ion energy content increases, the footprint stemming from the use phase will increase. Nevertheless, given the high charge/discharge efficiency and its high cyclability, it can be expected that the novel technology would be competitive with some of the current technologies used for stationary applications, including environmental performance terms. Numerical values of the batteries’ production phase for eight impact categories can be found in Table S25 and Figure S7.

#### 4.1.1. Influence of the Energy Source

As technology develops and evolves, the energy intensity associated with the production and its embodied carbon emissions is expected to decrease, leading to reduced pressure on the environment. Considering these two aspects, the undertaken sensitivity analysis explores the influence of the energy source on the environmental impacts embodied in the production of the batteries. The sensitivity analysis covers two scenarios: (1) Baseline scenario: The cells’ manufacturing features the same conditions as were modelled in this study; and (2) Clean production scenario: The assembly of both chemistries uses 100% PV energy. 

**Figure 5:**
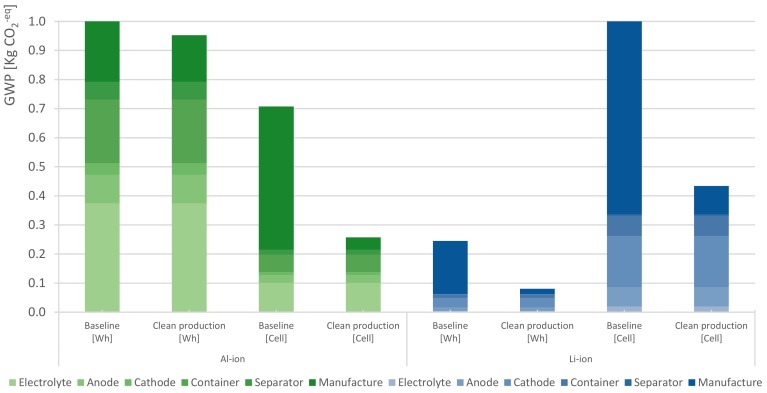
18650-cell’s sensitivity analysis. The baseline scenarios are based on the current environmental contribution of each chemistry for the corresponding functional unit. The clean production scenario assumes that the source of the energy used in the manufacturing phase is solar photovoltaic (PV). According to the simulation, Li-ion cells can cut 67% of their carbon footprint, while the Al-ion battery could reduce 64% of their GWP contribution.

Figure 5 shows the sensitivity analysis results for the GWP impact category for both chemistries. On a per-cell basis, the results show that the use of PV energy in the assembly process would drop carbon emissions from 0.7 to 0.26 kg CO_2_^−eq^ for the Al-ion chemistry and from 1.3 to 0.43 kg CO_2_^−eq^ for the Li-ion during the manufacturing phase. This means that in both scenarios, the production of one Al-ion cell is less carbon-intensive than the Li-ion cell. However, on an energy storage basis, the production of Li-ion cells has a significantly lower carbon footprint compared to the Al-ion chemistry. Indeed, the clean production of 1 Wh storage capacity would fall from 2.6 to 0.96 kg CO_2_^−eq^ in the case of the Al-ion cell, while for the reference cell, it would drop from 0.25 to only 0.09 kg CO_2_^−eq^. 

In short, the sensitivity analysis displays the potential benefits of using renewable energies in the assembly stage. Furthermore, given the environmental friendliness of the energy source, the assembly phase becomes less carbon-intensive relevant in the cell’s lifecycle, bringing the materials’ refinery and the electrolyte synthesis as more relevant stages in carbon emissions terms.

#### 4.1.2. Influence of the Data Resolution

Throughout the study, the energy intensity was identified as the most uncertain value. Furthermore, the results found that the energy consumption throughout the manufacturing phase is the most impactful factor for the environmental profile of the cells. In addition, in the literature, a wide spectrum of energy intensities was assumed for the production phase of batteries. Thus, a sensitivity analysis was performed regarding the energy intensity required for the manufacturing phase to test the robustness of the results in response to the influence of the energy source and to reduce the ambiguity of the study. Thus, the uncertainty analysis explores how the results could be affected by the differences in the energy intensity supplied. For this analysis, three different scenarios were compared. The first scenario undertakes the current energy intensity of the Li-ion cell production (34 kWh/kg). The second scenario considers a 30% reduction in energy usage in relation to Li-ion production. This assumption is based on the absence of a dry room in the manufacturing process which is the same energy intensity used to model the novel cell production. Finally, the third scenario assumes that improvements within the manufacturing phase exhibit a substantial reduction to 10 kWh/kg. Hence, with the three different energy intensities assumed, the GHG impacts are framed within a reasonable spectrum. 

**Figure 6:**
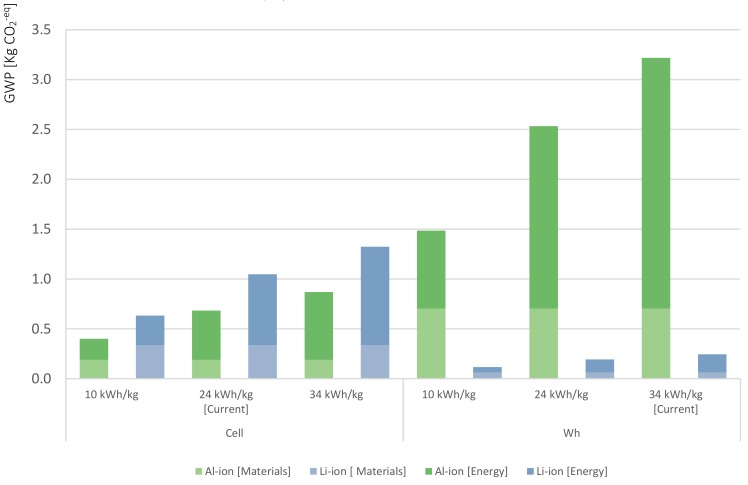
18650-cell’s uncertainty analysis. Three scenarios are modelled: the first scenario undertakes the current energy intensity of the Li-ion cell production (34 kWh/kg). The second scenario considers a 30% reduction in energy usage in relation to Li-ion production (24 kWh/hr). The third scenario assumes that improvements within the manufacturing phase exhibit a substantial reduction to 10 kWh/kg. The results emphasize the importance and relevance of the cells’ manufacturing energy efficiency and their direct correlation with the GWP contribution.4.2. Recycling Footprint

Figure 6 shows the absolute contribution to the GWP by the two chemistries in each of the three scenarios modelled and for both functional units. Since the energy usage alternatives were modelled only for the batteries’ assembly, the environmental impact from the components manufacturing were the same for all the scenarios (bars tagged as Al-/Li-ion [Materials]). The results suggest that on a per-Wh of energy storage basis, the production of the Al-ion cells cannot compete with the reference cells, not even in the lowest energy intensity scenario. On a per-cell basis, the picture is favourable for the Al-ion chemistry. That is, substantial carbon savings are found for all three scenarios due to the lower carbon footprint of its components and lower energy usage for the manufacturing of one cell. The simulation illustrates that the impacts are directly proportional to the energy intensity of the manufacturing process and the importance of the correlation of the energy storage capacity per cell with a lower environmental impact. 

**Figure 7:**
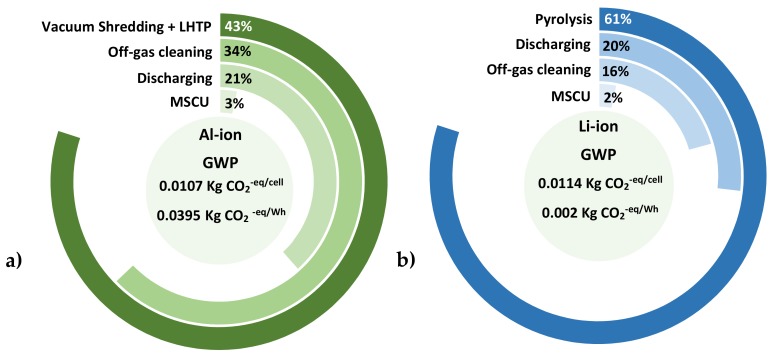
Comparison of the GWP contribution by the Al-ion’s (**a**) and Li-ion’s (**b**) recycling processes. Figure (**a**) shows the Al-ion recycling process with the total impact of 0.0107 kg of CO_2_^−eq^ in a cell basis and 0.0395 kg of CO_2_^−eq^ in a storage capacity basis. The main contributor, in this case, is the vacuum-shredding + LTHP. Figure (**b**) presents the Li-ion recycling process. This process performs poorer in comparison with the Al-ion recycling process due to pyrometallurgical operations. In this case, the total GWP contribution was estimated to be 0.0114 kg of CO_2_^−eq^ in a cell basis and 0.002 kg of CO_2_^−eq^ in a storage capacity basis.

Figure 7a presents the contribution of each of the Al-ion recycling phases to the total carbon equivalent emissions. Analogously, Figure 7b breakdowns the Li-ion’s recycling stages and their corresponded input to the GWP indicator. Similar to the environmental profile obtained in the production phase, the Al-ion cell has a better environmental performance on a per-cell basis while the Li-ion surpasses the novel technology in a per-Wh of energy stored basis. For the Al-ion cell, the breakdown of the impact shows that the main contributor to GHG emissions is the VS + LHTP stage, which contributes to 43% of the total carbon emissions. Its significant contribution can be attributed to the operation of a vacuum room, which is highly energy-intensive. The off-gas cleaning process is the second most important contributor and represents 34% of the carbon emissions. Its impacts are allocated to upstream activities related to the production of the active carbon used to filter the organic compounds from the thermal process. For the Li-ion’s recycling process, the pyrometallurgical operations are the major contributor causing 61% of the total emissions due to the high energy requirements. The second most intensive stage of this chemistry is the discharging stage, which contributes to 20% of the carbon emissions. Its carbon loads are assigned to a large amount of brine used in the process.

Essentially, the novel recycling process designed by ACCUREC shows that reductions of approximately 6% can be achieved if pyrometallurgical operations are substituted for a vacuum shredding + LHT process. Numerical values of the EoL stage for eight impact categories can be found in Table S26 and the relative contribution of each step is depicted in Figure S7.

## 5. Conclusions

This study performed a comprehensive and transparent LCA to assess the environmental characterization of the novel Al-ion cell in a 18650 format. To evaluate the environmental profile of the novel technology, this study opted to compare its environmental performance with the state-of-the-art of technologies in current use, i.e., a Li-ion NMC battery, using a cradle-to-grave approach. Moreover, to provide an impartial assessment, two functional units were chosen which allowed to exanimate the novel battery’s environmental performance from two different perspectives.

On one hand, the results point out that on a per-cell basis, the Al-ion chemistry is outstanding over the reference technology. The main environmental advantages spring from the lack of copper and NMC pastes, the use of materials with a lower carbon load, such as graphite, and the avoidance of using a dry room during the manufacturing phase. On the other hand, in a per energy content basis, the main disadvantage of the Al-ion technology was found to be its low energy density. Essentially, on an energy content basis, the results demonstrate that the higher the energy density of the cell is, the lower the environmental impacts per energy unit, which makes the reference technology the clear winner for this comparison.

Analogous to the research performed by Longo et al. [21], Spanos et al. [20] and Vandepaer et al. [22], this study found that, regardless of the functional unit and technology, the production phase has the highest contribution to the GWP impact category. Specifically, the assembly process contributes the most due to the high carbon intensity of the electricity mix used in the process. In general, it was found that the assembly process during the production phase is the most carbon-intensive stage. Ergo, a more sustainable Al-ion battery development can be achieved if the energy consumption during the battery’s assembly relies mainly on renewable sources, as was shown by the sensitivity analysis. Furthermore, the contribution analysis identified the electrolyte used in the Al-ion battery as a critical component due to the considerable high amount used and its material composition. Therefore, the authors advise exploring potential substitutes to the aluminium chloride in order to decrease the use of high carbon-intensive materials. Furthermore, substantial carbon emissions can be avoided in the end-of-life stage if the electrolyte is recovered and reused instead of being disposed after been captured by active carbon. The novel Al-ion technology is still in a low TRL. Nevertheless, through the ALION project, substantial improvements were achieved. Thus, it is expected that its performance can be increased and eventually overcome the current performance of modern batteries. From a mere environmental perspective, an Al-ion 18650-cell can surpass a Li-ion battery when a density of 60 Wh/kg and 5000 cycles are achieved. 

Finally, at the end of the ALION project, the novel battery was identified to have a high-power property, which opens new opportunities to cover different applications than only energy storage. This means that the consequential implications to assess its environmental characterization would change. Therefore, the authors recommend exploring other applications where the intrinsic characteristics of the novel technology can position it as a competitive option in the market. 

## Supplementary Materials

The following are available online at https://www.mdpi.com/1996-1944/12/19/3270/s1. Tables S1–S24 contain the compiled first two tiers of the inventory used to assess the environmental characterization of the novel Al-ion battery and the reference Li-ion battery (Tables S1–S24). Tables S25–S28 present the complete numerical values resulting from the production and recycling phase for eight impact categories (Tables S25–S28). Figure S1, 18650 Al-ion cell composition by components and materials, Figure S2, 18650 production process. The black boxes represent background products that are further used by the foreground products (white boxes) while the rhombuses represent unit operations, Figure S3, recycling steps designed by ACCUREC for the Li-ion cell, Figure S4, 18650 Li-ion cell composition by components and materials, Figure S5, flowchart for the production of the 18650 Li-ion battery cell. Figure S6, recycling steps designed by ACCUREC for the Li-ion cell, Figure S7, production phase contribution analysis for eight impact categories of Li-ion and Al-ion 18650 cells, Figure S8, contribution analysis for eight impact categories of the Al-ion recycling process. Furthermore, the sensitivity and uncertainty numerical results are displayed at the end of the section.
