# Correction: Salgado Delgado, M.A., et al. Comparative Life Cycle Assessment of a Novel Al-Ion and a Li-Ion Battery for Stationary Applications. *Materials* 2019, *12*, 3270

**DOI:** 10.3390/ma12233893

**Published:** 2019-11-25

**Authors:** Mario Amin Salgado Delgado, Lorenz Usai, Linda Ager-Wick Ellingsen, Qiaoyan Pan, Anders Hammer Strømman

**Affiliations:** 1 Industrial Ecology Program, Norwegian University of Science and Technology, E1-Høgskoleringen 5, 7491 Trondheim, Norway; 2 ACCUREC Recycling GmbH, Bataverstraße 21, DE-47809 Krefeld, Germany; qiaoyan.pan@accurec.de

The authors wish to make the following corrections to this paper [1]: We have omitted the contribution of the co-author Dr. Linda Ager-Wick Ellingsen.

The authors would like to apologize for any inconvenience caused to the readers by this change.
