# Supplementary material for: Comparative Life Cycle Assessment of a Novel Al-Ion and a Li-Ion Battery for Stationary Applications
Source: Materials (Basel). 2019 Oct 8;12(19):3270. doi: 10.3390/ma12193270 (PMC6803941; doi:10.3390/ma12193270)
Supplement: Supplementary file 1 [file materials-12-03270-s001.pdf]

Article

Comparative Life Cycle Assessment of a Novel Al-ion and a Li-ion battery for stationary applications

Mario Amin Salgado Delgado [1,*], Lorenzo Usai [1], Qiaoyan Pan [2] and Anders Hammer Strømman [1]

[1] Industrial Ecology Program, Norwegian University of Science and Technology, Trondheim, Norway, E1-Høgskoleringen 5, Trondheim 7491, Norway; lorenzo.usai@ntnu.no (L.U.); anders.hammer.stromman@ntnu.no (A.H.S.)
[2] ACCUREC Recycling GmbH, Bataverstraße 21, DE-47809 Krefeld, Germany; qiaoyan.pan@accurec.de
* Correspondance: amin.salgado@ntnu.no; Tel: +47-967-06-674

Received: 8 August 2019; Accepted: 27 September 2019; Published: date

Summary

This supplementary material is split into two sections. Section 1 contains the compiled first two tiers of the inventory used to assess the environmental characterization of the novel Al-ion battery and the reference Li-ion battery. Section 2 presents the complete numerical values resulting from the production and recycling phase for eight impact categories. Furthermore, the sensitivity and uncertainty numerical results are displayed at the end of the section.

Inventory

1.1. The Al-ion 18650 cell

As presented by Ellingsen et al. [1], Al-ion batteries can be made of a combination of different types of materials. Particularly, the consortium converged to use pure aluminium for the anode and pyrolytic graphite for the cathode. Regarding the electrolyte an [EMIM][TFSI] 0.5M $AlCl_3$ solution is proposed. The data is based on the bill of material (BOM) for the 18650 format and the weight of the battery is 29.3 grams. The battery material composition by components and their corresponding materials is shown in Figure S1.

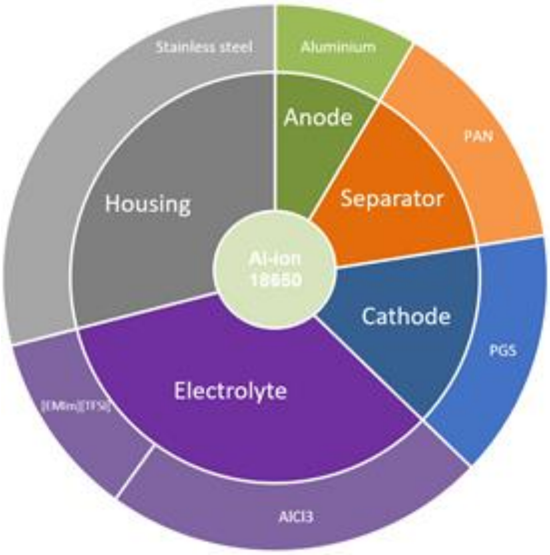

Figure S1. 18650 Al-ion cell composition by components and materials.

The 18650 cell consists of five main components: the cell canister, the separator, the electrolyte, the anode, and the cathode. To build the battery's inventories, both generic datasets and novel datasets were compiled specifically for this study. The novel datasets created are primarily based on data provided by the consortium and literature review. As such, partners in the **ALION** project

provided the data needed to build the 18650 Al-ion battery cell inventory. The following sections present the inventory compilation for the manufacturing and recycling process.

1.1.1. Al-ion Production

Our partner and manufacturer of the electrolyte shared the electrolyte's production process data, and the synthesis steps to develop the electrolyte's inventory. Regarding the other four battery's components, our partner in charge of the battery assembly provided the BOM for the prototype cell. Figure S2 illustrates the Al-ion battery production process. As it can be observed, the most complex production line is for the electrolyte. This is due to the need for breaking down all the synthesis steps to achieve a more accurate dataset. In the flowchart, the black boxes represent background products that are further used by the foreground products (white boxes) while the rhombuses represent unit operations. Please note that the convention for life cycle inventories involves the listing of both materials use and material processing activities. Both might be listed in kilograms of materials and may be misinterpreted as double counting.

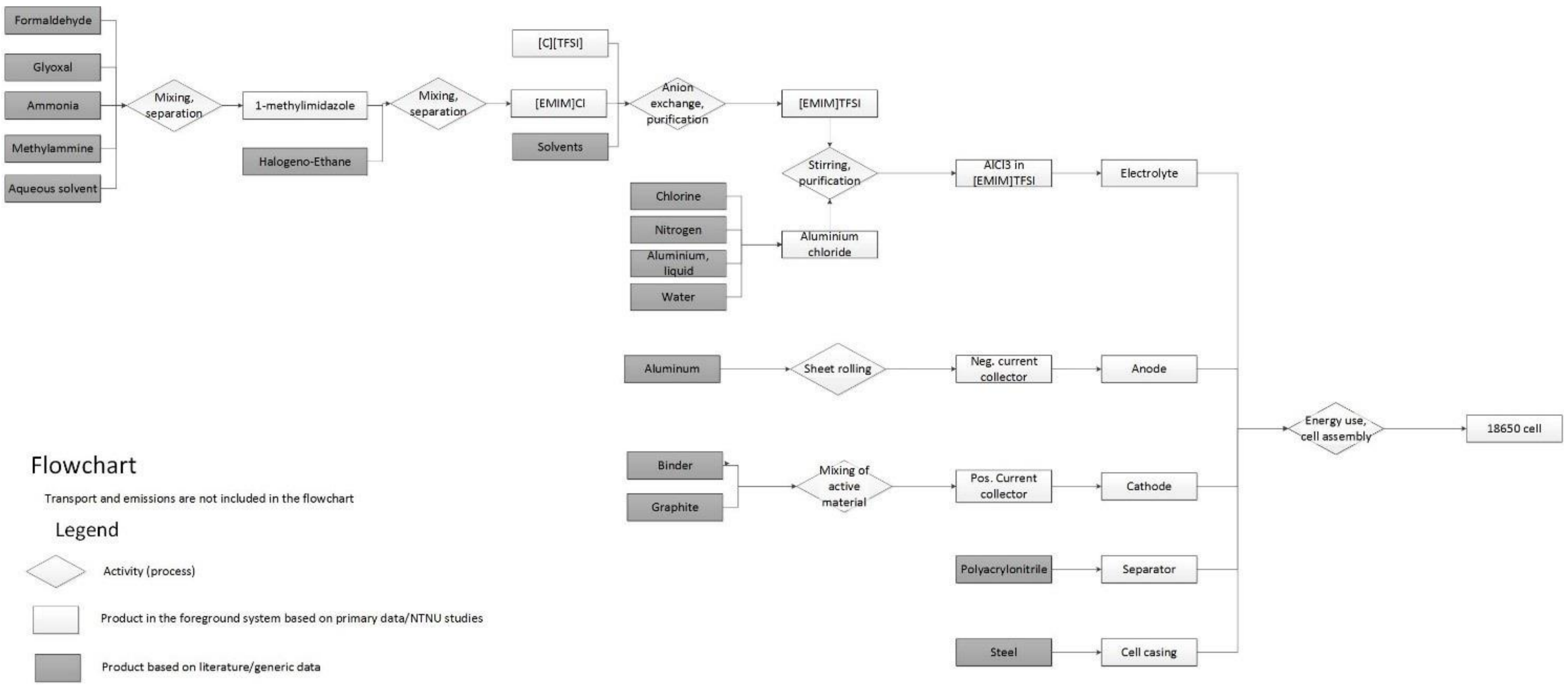

Figure S2. 18650 production process. The black boxes represent background products that are further used by the foreground products (white boxes) while the rhombuses represent unit operations.

Table S1 shows the first-tier inventory of the Al-ion 18650 and it is broken down into components, energy, and infrastructure requirements used in the manufacturing phase. Table S2–S6 show the second-tier inventories compiled for each of the Al-ion 18650 components. Simultaneously, we explain the corresponding process that takes place and the assumptions (if any). In addition to the materials composition, the inventories consider factors such as infrastructure, energy, and transport involved along the production chain. Transport requirements and infrastructure assumptions are estimated using in-house average data for the corresponding supply chain.

Table A1. Al-ion 18650 cell's composition.

Item		Share	Amount	Unit	Reference Process
**Functional unit**	Al-ion cell		0.029	kg	
**Components**	Anode	9%	0.003	kg	See Table S2
	Separator	14%	0.004	kg	See Table S3
	Cathode	15%	0.004	kg	See Table S4
	Cell canister	29%	0.009	kg	See Table S5
	Electrolyte	34%	0.010	kg	See Table S6
**Energy & processes**	Electricity requirements		20.67	kWh	*electricity mix, medium voltage/ SK**
**Infrastructure**	Cell manufacturing infrastructure		1.9E-08	p	*facilities precious metal refinery/ SE*

1.1.1.1. Anode

With the 9% weight share, the anode is the lightest component of the 18650 cell. The anode, by design, is made of high purity aluminium foil. Therefore, we modelled primary aluminium production and assumed a sheet rolling process using the European context.

Table S2. Al-ion 18650 anode's inventory.

	Item	Share	Amount	Unit	Reference Process
**Functional unit**	Anode		0.003	kg	
**Materials**	Aluminium foil		0.003	kg	*aluminum production, primary, ingot/EU27 & EFTA*
**Processes**	Sheet rolling		0.003	kg	*sheet rolling, aluminum/ RER*
**Transport**	Rail		0.002	tkm	*market for transport, freight train/EU without CH*
	Lorry >32t		0.001	tkm	*transport, lorry >32t, EURO3/ RER*
**Infrastructure**	Facility		1,5E-10	p	*aluminum casting, plant/ RER*

1.1.1.2. Separator

From the different materials tested, a mix of acrylonitrile and methacrylate was chosen as the material that meets most of the requirements with good mechanical properties. The mixture results in polyacrylonitrile (PAN). For modelling the PAN synthesis, the materials and energy requirements involved were taken from Johnson's work [2]. For the 18650-type, the separator counts for the 14% of the cell's weight, and we assumed that an extrusion process for plastic film was used to manufacture the separator.

Table S3. Al-ion 18650 separator's inventory.

Item		Share	Amount	Unit	Reference process
**Functional unit**	Separator		0.0042	kg	
**Materials**	Acrylonitrile	95%	0.0040	kg	*acrylonitrile/market for acrylonitrile/GLO*
	Methyl methacrylate	5%	0.0002	kg	*market for methyl methacrylate/GLO*
**Energy & processes**	Natural gas		0.0039	m3	*market group for natural gas, high pressure/EU without CH*
	Electricity		0.0059	kWh	*market group for electricity, medium voltage/GLO*
	Extrusion*		0.0042	kg	*extrusion production, plastic film/RoW*
**Transport**	Rail		0.2	tkm	*market for transport, freight train/EU without CH*
	Lorry >32t		0.1	tkm	*transport, lorry >32t, EURO3/ RER*

**Infrastructure**	Facility	7.0E-10	p	*plastics processing factory/ RER*

1.1.1.3. Cathode

The cathode is mainly made of pyrolytic graphite (PG) and accounts for 15% of the total cell's weight. Table S4 breaks down the composition of the cathode based on primary data provided BY our partner in charge of the battery's assembly. For this element, it was assumed the use of glue and solvents to keep the graphite powder in the form of a foil. Due to the non-existence of specific datasets to model the pyrolytic graphite, the battery-grade graphite dataset is considered to give representative results.

Table S4. Al-ion 18650 cathode's inventory.

**Item**		**Share**	**Amount**	**Unit**	**Reference process**
**Functional unit**	Cathode		0.0043	kg	
**Materials**	CMC	2%	0.0001	kg	*CMC, powder, at plant/ RER*
	PAA	2%	0.0001	kg	*acrylic acid, at plant/ RER*
	Graphite	96%	0.0041	kg	*graphite, battery grade, at plant/ CN*
**Transport**	Rail		0.21	tkm	*market for transport, freight train/Europe without CH*
	Lorry >32t		0.10	tkm	*transport, lorry >32t, EURO3/ RER*
**Infrastructure**	Facility		4E-10	p	*chemical plant, organics/ RER*

1.1.1.4. Cell canister

The cell canister, or housing, is the component that covers the whole cell. According to primary sources, the canister shares 29% of the total cell's weight and is made of steel. To model the cell canister, the chromium steel market and a generic steel manufacturing process were used to represent the component's supply chain.

Table S5. Al-ion 18650 canister's inventory.

**Item**		**Share**	**Amount**	**Unit**	**Reference process**
**Functional unit**	Cell canister		0.0085	kg	
**Materials**	Steel	100%	0.0085	kg	*chromium steel 18/8/market for steel/GLO*
**Energy & processes**	Steel production		0.0085	kg	*steel product manufacturing, average metal working/ RER*
**Transport**	Rail		0.2	tkm	*market for transport, freight train/EU without CH*
	Lorry >32t		0.1	tkm	*transport, lorry >32t, EURO3/ RER*
**Infrastructure**	Facility		4.6E-10	p	*metal working factory/ RER*

1.1.1.5. Electrolyte

Finally, the electrolyte is the heaviest component, accounting for 34% of the cell's weight. The electrolyte's synthesis starts by mixing five solutions, which after reacting to each other undergo several separation processes (distillation, extraction, and crystallization) to obtain 1-methylimidazole [3]. Later, the aromatic compound is mixed with halogenated ethane and recrystallized to produce [EMIM]Cl, which in the presence of [C][TFSI] and organic solvents are subjected to a separation process (anions exchange) and later purified to acquire the [EMIM]TFSI [4]. Analogously, the aluminium chloride ($AlCl_3$) can be synthesized by an exothermic reaction of aluminium with chlorine gas [5]. Finally, the [EMIM]TFSI and $AlCl_3$ are mixed to a weight ratio of approximately 1:2 and purified to get [EMIM][TFSI] 0.5M $AlCl_3$ which is used as the electrolyte for the 18650-type cell [4].

To model this section of the LCI, the data provided by our project partner was combined with a literature review where several sources were taken into account for the final inventory compilation [6–8]. Furthermore, the fragmentary data required different assumptions. For example, we consider trifluoromethanesulfonic acid (i.e., triflic acid; TFSA) as a proxy instead of TFSI. Moreover, we assume that a halogen is used to stimulate the anion exchange between EMI and TFSI. Lastly, where

minor data was missing throughout the synthesis of the electrolyte, we opted to utilize generic processes, such as *organic solvents*.

Table S6. Al-ion 18650 electrolyte's inventory.

Item		Share	Amount	Unit	Reference process
**Functional unit**	AlCl3 in [EMI][TFSI]		0.0100	kg	
**Materials**	Aluminum chloride	67%	0.0067	kg	
	[EMI][TFSI]	33%	0.0033	kg	
**Energy & processes**	Heat		0.030	MJ	*heat/steam production in chemical industry/RoW*
**Transport**	Rail		0.60	tkm	*freight train/market for transport, freight train/EU without CH*
	Lorry >32t		0.10	tkm	*transport, lorry >32t, EURO3/ RER*
**Infrastructure**	Facility		4.0E-10	p	*chemical plant, organics/ RER*
**Waste**	Spent solvent		4.0E-10	p	*spent solvent mixture/market for spent solvent mixture/GLO*
**Emissions**	Heat		0.030	MJ	*heat, waste/air/unspecified*

1.1.2. End of life

Figure 7 illustrates a flowchart of the recycling process along with its main phases. The process is a combination of mechanical dismantling and separation, electrochemical, and thermal treatment. The process consists of the following three main operations plus one cleaning system: discharging, vacuum shredding and LTHP, mechanical separation and off-gas cleaning. In addition, the diagram aims to provide a mass balance using a calculation base of 100 kg of spent batteries and to display from which recycling phase each component of the battery cell can be recovered.

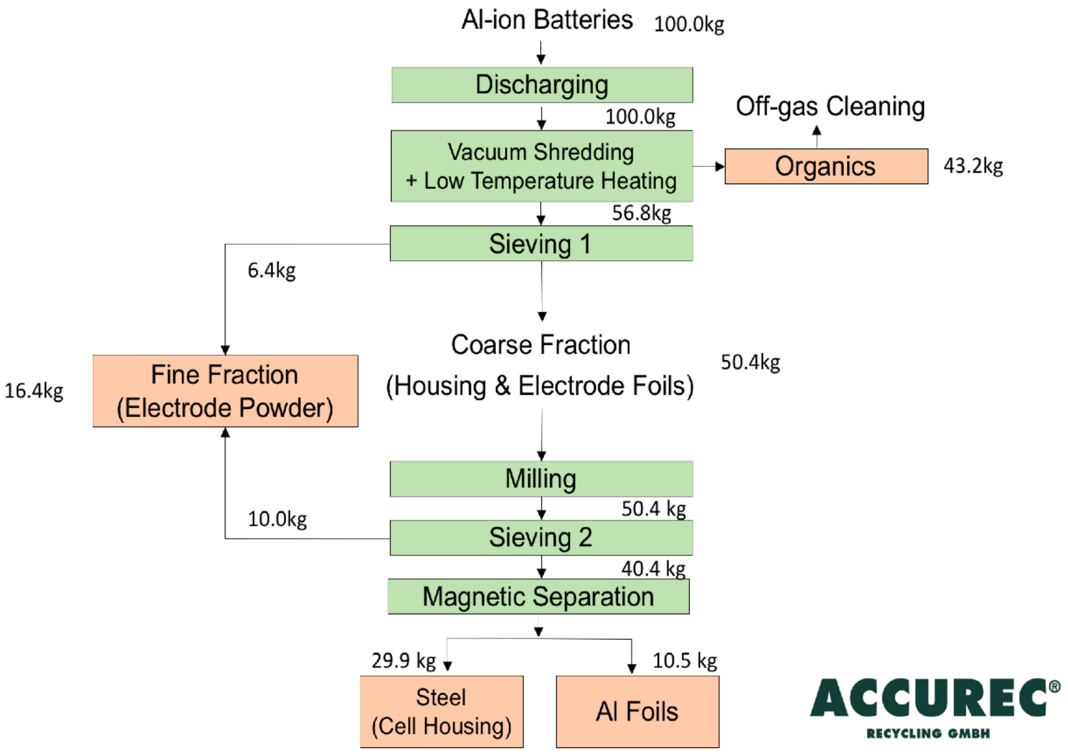

Figure S3. Recycling steps designed by ACCUREC for the Li-ion cell [9].

The inventory compiled for the assessment of the EOL treatment takes into account in each step the electricity required by the machinery and for heating, the machinery used (modelled as a mass of steel), and the chemicals and materials used along the recycling stages. Table S7 lists the three main stages within the recycling process (first tier) i.e., battery discharging, vacuum shredding + LTHP and the mechanical separation processes. Tables S8-11 present the second-tier of the recycling process

inventory. The functional unit used to simulate the whole process was one metric ton of Al-ion 18650 batteries, but the results where further harmonized giving the impact per cell, or per watt-hour, which are the two functional units used for the previous phases. Since the brine used for the discharging phase can be reused for an undefined amount of times, only a small share of the impact was given to a battery. Therefore, for linking a small share of the impact to each battery, it was assumed that the solution is used for eight years with an annual production capacity of 2 million metric tons.

Table S7. Al-ion 18650 end-of-life (EOL) process composition.

Item		Flow	Unit	Reference process
**Functional unit**	EOL Al-ion 18650	1000	kg	
**Process steps**	Discharging	2.9E-07	pc	See Table 8
	Vacuum shredding + low-temperature heating	1	pc	See Table 9
	Off-gas cleaning system	1	pc	See Table 10
	Mechanical separation combined unit	1	pc	See Table 11
**Infrastructure**	Cell manufacturing infrastructure	1	pc	*precious metal refinery construction/SE*

1.1.2.1. Discharging

For safety reasons, the first step within the recycling process is to ensure the full discharge of the batteries. In this stage, the cell units are immersed in brine for two weeks, specifically in a solution made of potassium hydroxide (KOH) and water. The result of this stage is a discharging liquid (brine), which can be reused in the same process for many more cycles and the discharged batteries ready to continue the recycling process. The brine acts as an electrolyte, and therefore electricity is also used in this step (Table S8).

Table S8. Al-ion discharging process inventory.

Item		Flow	Unit	Reference process
**Functional unit**	Discharging	1	pc	
**Materials**	KOH flakes	26.25	kg	*market for potassium hydroxide/GLO*
	H_2O	1023.75	kg	*water production and supply, decarbonised/RER*
**Energy & processes**	Electricity for discharging	3.33	kWh	*market for electricity, medium voltage/DE*

1.1.2.2. Vacuum shredding + low-temperature heating

The second stage is a shredding process that operates in a vacuum to avoid exposing the cells' contents to oxygen, water vapour, and other impurities present in the atmosphere, reducing the likelihood of internal short circuits, which can be violent in contact with oxygen. Simultaneously, a thermal treatment known as LTHP operating at 400°C takes place. As an output of this stage, the electrodes and canister materials are separated from the organic compounds (i.e., the electrolyte and the separator). Next, the electrodes and canister materials continue the recycling process, while the organic compounds are retained in a cleaning system, as described below.

Table S9. Al-ion vacuum shredding and low-temperature heating process (LTHP) inventory.

Item		Flow	Unit	Reference process
**Functional unit**	Vacuum shredding + LHTP	1	pc	
**Energy & processes**	Electricity for shredding	18.50	kWh	*market for electricity, medium voltage/DE*
	Electricity for heating	172.22	kWh	*market for electricity, medium voltage/DE*

1.1.2.3. Off-gas cleaning of the electrolyte

The off-gas cleaning system is an auxiliary process and primarily directed at capturing the emissions generated during the heat treatment. The system consists of an adsorbent (activated

carbon), which reduces the air emissions of particulate matter, odours, and VOCs. Notably, the recycling process has not been designed to recover any organic compounds, so after the adsorbent has captured these compounds, the activated carbon will be disposed as hazardous waste. To model this stage, the inventory compiled takes into account the production of the activated carbon and the treatment of the consequential waste generated in the cleaning operation.

Table S10. Al-ion off-gas cleaning process inventory.

Item		Flow	Unit	Reference process
Functional unit	Off-gas cleaning	1	kg	
Materials	Crude coal	36	kg	*hard coal/market for hard coal/AU*
	Water for activation	148	kg	*water production and supply, decarbonised/RER*
Energy & processes	Electricity	1.8	kWh	*market for electricity, medium voltage/DE*
	Steam heating	13.3	MJ	*heat production, natural gas, at industrial furnace low-NOx >100kW/EU without Switzerland*
Infrastructure	Facility	3.2E-10	pc	*market for industrial furnace, coal, 1-10MW/GLO*
Waste	Hard coal ash	−0.17	kg	*treatment of hard coal ash/CH*

1.1.2.4. Mechanical separation combined unit

Once that the organic compounds have been separated and the rest of the battery has been cut into ribbons, the materials enter to a mechanical separation unit where a first sieving process separate the coarse fraction, which after this stage accounts for the 50% battery's weight. Later, these materials are milled and sieved again before using a magnet to attract the magnetically susceptible material in the battery. In the two-sieving process, it is expected that the fine fraction recovered is composed of the cathode material (i.e., PG. The PG is planned to be used as a reducing agent.

The inventory created to model the last stage of the recycling process considers the use of machinery and the energy consumed to operate it. During an LCA of a system, outputs such as the recovery of materials in a recycling process can be treated differently according to the allocation system chosen. For the second case assessed, where the recovered materials are accounted for, we chose the so-called allocation at the point of substitution (APOS), where the outputs can be defined as allocable by-products, recyclable materials or wastes [10]. As a result, the graphite, steel and aluminium recovered are considered recyclable materials, meaning that a benefit is allocated in the recycling process due to the avoided production of the equivalent material in a different process. Thus, the EOL phase gets a negative value from this stage. Table 15 shows the baseline inventory, where neither benefits nor burdens are allocated to the recycling process, for the recovery.

Table S11. Al-ion mechanical separation combined unit's inventory.

Item		Flow	Unit	Reference process (proxy)
Functional unit	Mechanical separation combined unit	1	pc	
Machinery	Steel	1180	kg	*steel, low-alloyed/market for steel, low-alloyed/GLO*
Energy	Electricity for shredding	18.50	kWh	*market for electricity, medium voltage/DE*
Outputs	Electro powder	0	kg	
	Steel (cell housing)	0	kg	*market for scrap steel/GLO*
	Aluminium foil	0	kg	*market for waste aluminium/GLO*

1.2. The Li-ion Battery Cells

To understand whether the novel technology can bring some benefits under an environmental perspective, the results must be compared with a chosen benchmark. For the reference cells, we

assumed a $Li(Ni_{0.45}Mn_{0.45}Co_{0.10})O_2$ chemistry. The data is based on the bill of material (BOM) for the 18650 format and the weight of the battery is 43 grams. The Li-ion cell consists of the same five components named in section 1.1. This is the cell canister, the separator, the electrolyte, the anode, and the cathode. To production phase inventory was built with in-house data [11], while for the end-of-life phase our project partner **ACCUREC** contributed with the necessary data to model the recycling process. The following sections present the inventory compilation for the manufacturing and recycling process. The battery material composition by components and their corresponding materials is shown in Figure S4.

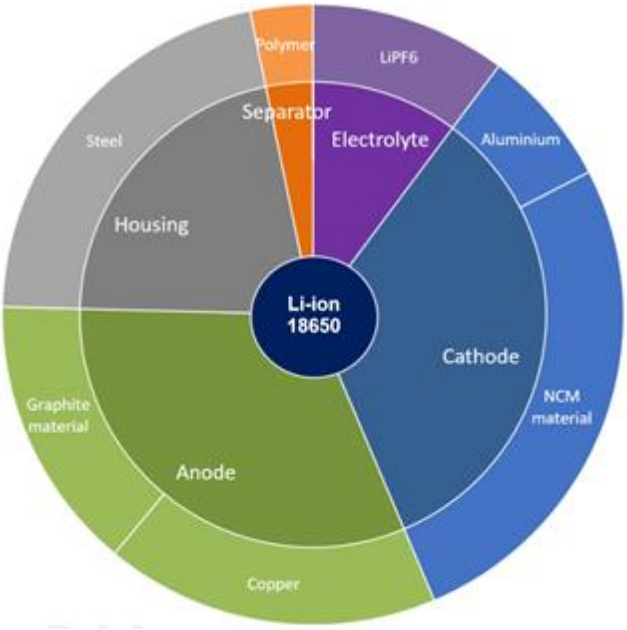

Figure S4. 18650 Li-ion cell composition by components and materials.

1.2.1. Li-ion Production

Figure S5 presents the production process of the reference cell. The flowchart distinguishes between products (rectangle) and activities (rhombus). It also differentiates the foreground system (white background) with data compiled specifically for this study from the background system (grey background) where we use the *ecoinvent* 3.2 database as provider of information about the environmental intensity of the materials and processes accounted for. In the following tables, we dig deeper into the LCIs modelled for each component, showing its weight, its composition, and the ecoinvent datasets assumed for each material or process used. Simultaneously, the assumptions and simplifications done throughout the compilation of the inventories are explained.

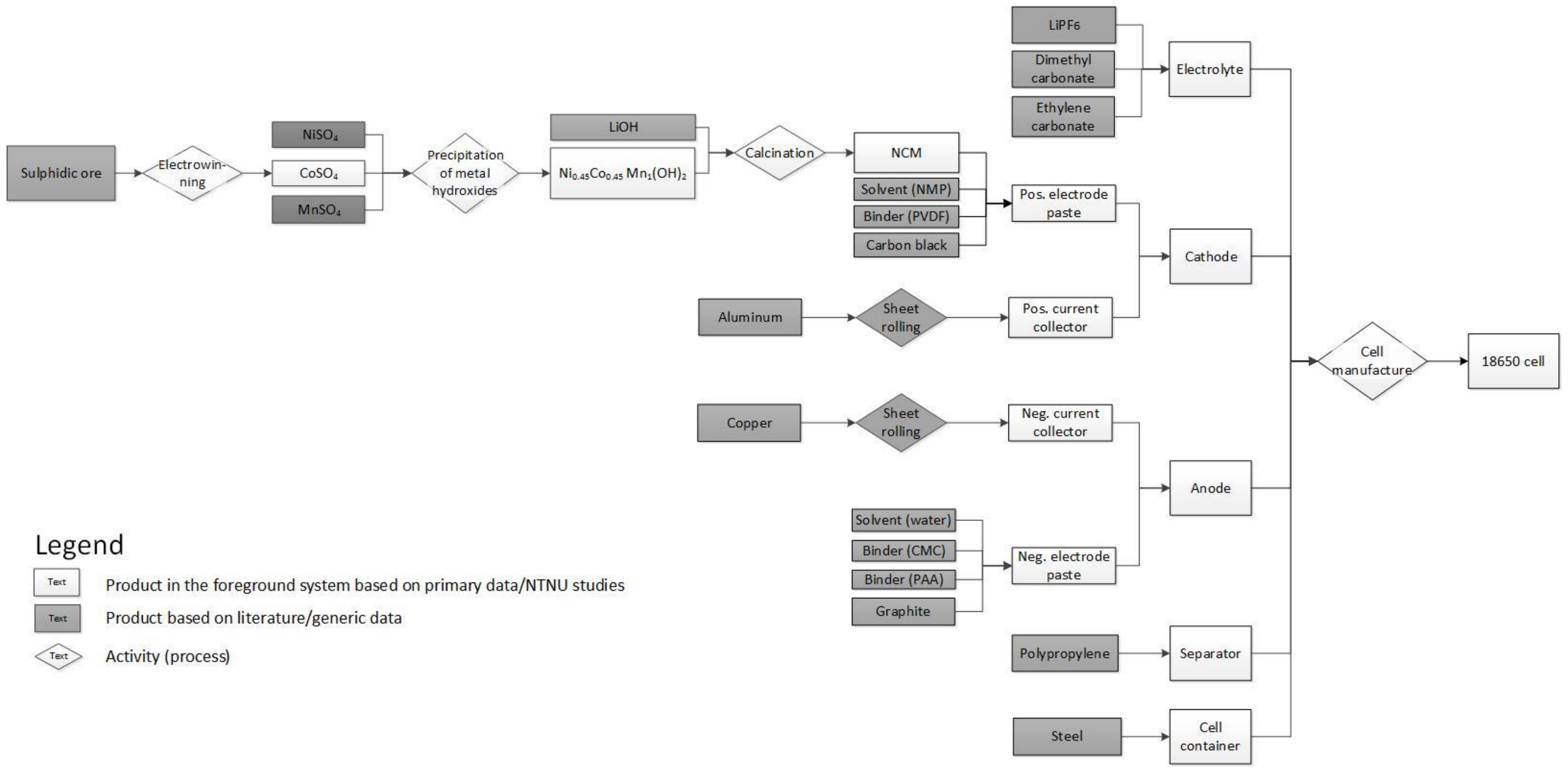

Figure S5. Flowchart for the production of the 18650 Li-ion battery cell.

A BOM provided by Grenland Energy [13] laid the foundation for the cradle-to-gate inventory compilation. Table S13 shows the first-tier inventory of the Li-ion NCM cell. From Table S14 to S18 we present the inventories for each of the Li-ion cell components (second-tier). Tables S19-S24 show the inventory compilation regarding the recycling phase. The energy required for the cell manufacture of the 18650 cell is around 33.4 kWh/kg [13]. The cells modelled in this inventory are produced in South Korea, and consequently, the Korean electricity mix was assumed. For all the components, the transport was calculated using the standard transport distances [11]. Finally, the metals have been modelled as primary materials coupled with the average metal working process; thus, the production of the material and its preparation for application are considered. The mentioned processes are included in each table, and they will not be further discussed since the assumptions presented are valid for each case. The LCIs compiled for each component included in the battery will now be presented, ordered from the lightest to the heaviest.

Table S13. Li-ion 18650 cell's composition.

Item		Share	Amount	Unit	Reference process
**Functional unit**	Li-ion Cell		0.0431	kg	
**Components**	Separator	*3%*	0.001	kg	See Table S14
	Electrolyte	*10%*	0.004	kg	See Table S15
	Cell container	*21%*	0.009	kg	See Table S16
	Anode	*32%*	0.014	kg	See Table S17
	Cathode	*33%*	0.014	kg	See Table S18
**Energy**	Electricity requirements		33.40	kWh	*South Korean electricity mix*
**Infrastructure**	Cell manufacturing		1.9E-08	p	*facilities precious metal refinery/ SE*
**Transport**	Freight, rail		0.6	tkm	*transport, freight, rail/ RER*
	Lorry		0.1	tkm	*transport, lorry >32t, EURO3/ RER*

1.2.1.1. Separator

The separator is the lightest component of the battery cell, with a contribution of 3% to the total weight. The polyolefin separator modelled and used by Miljøbil Grenland is a mixture of polypropylene and polyethylene. Table S14 presents the separator's inventory.

Table S14. Li-ion 18650 separator's inventory.

Item		Share	Amount	Unit	Reference process
**Functional unit**	Separator		0.001	kg	
**Materials**	Propylene	*67%*	9.33E-04	kg	*propylene production/RoW*
	Polyethylene	*33%*	4.67E-04	kg	*polyethylene production, linear low density, granulate/RoW*
**Processes**	Extrusion		0.001	kg	*extrusion production, plastic film/RoW*
**Transport**	Freight, rail		0.2	tkm	*market for transport, freight train/Europe without Switzerland*
	Lorry		0.1	tkm	*transport, freight, lorry >32 metric ton, EURO3/RER*
**Infrastructure**	Facility		7.41E-10	p	*plastics processing factory/ RER*

1.2.1.2. Electrolyte

The electrolyte is a mixture of lithium hexafluorophosphate (LiPF6), dimethyl carbonate (DMC), and ethylene carbonate (EC). The amounts of DMC and EC are the same in the electrolyte, but because no data was found to produce DMC, the use of a double amount of EC was assumed.

Table S15. Li-ion 18650 electrolyte's inventory.

Item		Share	Amount	Unit	Reference process
**Functional unit**	Electrolyte		0.0044	kg	
**Materials**	LiPF6	*10%*	0.00044	kg	*market for LiPF6/GLO*
	Ethylene carbonate	*90%*	0.00396	kg	*ethylene carbonate production/CN*
**Transport**	Freight, rail		0.6	tkm	*market for transport, freight train/Europe without Switzerland*
	Lorry		0.1	tkm	*transport, freight, lorry >32 metric ton, EURO3/RER*
**Infrastructure**	Chemical Plant		4E-10	p	*chemical factory construction, organics/RER*

1.2.1.3. Cell canister

The cell canister is made of chromium steel, with a weight of almost 0.01 kg, it contributes to 20% of the total cell weight.

Table S16. Li-ion 18650 canister's inventory.

Item		Share	Amount	Unit	Reference process
**Functional unit**	Cell container		0.0092	kg	
**Materials**	Steel		0.0092	kg	*market for steel, chromium steel 18/8/GLO*
**Process**	Metal working		0.0092	kg	*metal working, average for chromium steel product manufacturing/RER*
**Transport**	Freight, rail		0.2	tkm	*market for transport, freight train/Europe without Switzerland*
	Lorry		0.1	tkm	*transport, freight, lorry >32 metric ton, EURO3/RER*
**Infrastructure**	Facility		4.58E-10	p	*metal working factory/ RER/ unit*

1.2.1.4. Anode

As a negative current collector, primary copper was assumed as high-purity metals are required in the cell to ensure good electrochemical performance. Most of the graphite material consists of graphite (97%), while PAA and CMC, at 1.6% of the total weight each, make up the remainder of the material. Due to a lack of data regarding the synthesis of PAA, acrylic acid was assumed instead. It was assumed that an aqueous solvent was used to slurry the graphite material for application to the copper foil in electrode production.

Table S17. Li-ion 18650 anode's inventory.

Item		Share	Amount	Unit	Reference process
**Functional unit**	Anode		0.014	kg	
**Component**	Negative current collector Cu	*55%*	0.008	kg	*copper/copper production, primary/RAS/kg*
	Negative electrode paste	*45%*	0.006	kg	*graphite, battery grade, at plant/ CN/ kg*

1.2.1.5. Cathode

The positive current collector is made of aluminium and contributes to 7.2% of the cell weight. Analogously to the negative current collector, also the positive current collector was assumed as made by 100% of primary material. The positive active material is the single heaviest material in the cell and contributes to 26% of the cell weight alone. The majority of the NCM material consists of NCM (94% by weight) while the PVDF binder (4.0%) and the conductive additive carbon black (2%) makes the rest of the weight. Because there is no *ecoinvent* process for PVDF, we assumed polyvinylfluoride (PVF) instead. The NMP solvent was used to slurry the NCM material for application onto the aluminium foil.

Table S18. Li-ion 18650 cathode's inventory.

Item		Share	Amount	Unit	Reference process
**Functional unit**	Cathode		0.0144	kg	
**Component**	Positive current collector Al	*22%*	0.003	kg	*primary, ingot/aluminium production/IAI Area 4&5*
	Positive electrode pastes NMC	*78%*	0.011	kg	*manganese sulfate/manganese dioxide production/GLO/kg*<br>*nickel sulfate/market for nickel sulfate/GLO/kg*<br>*soda, powder, at plant/ RER/ kg*

1.1.2. End-of-life treatment

The recycling process designed by **ACCUREC**, for the Li-ion cells, is composed of three main steps: complete discharging of the battery cells, pyrometallurgical, and a multi-step mechanical treatment. Figure S6 shows the processes involved and the recovered materials when recycling 100 kg of batteries. The recycling process can reach a recycling rate over 55%, and the only losses are related to the organics that cannot be recovered.

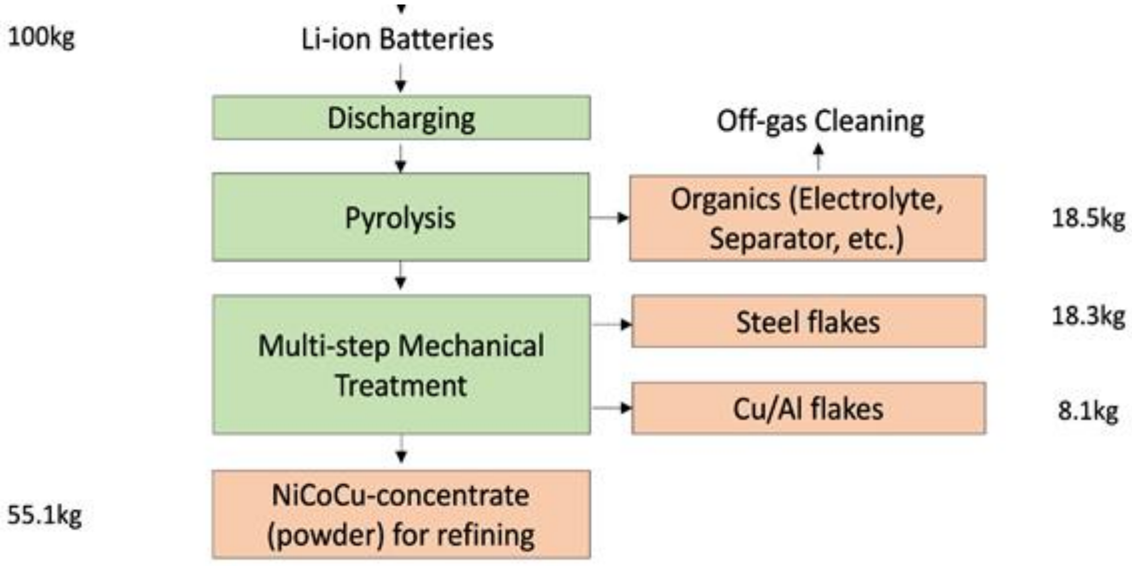

Figure S6. Recycling steps designed by ACCUREC for the Li-ion cell **[9].**

The LCIs for the EOL only considers the energy and materials needed for the entire recycling process. The main recycling steps are outlined in Table S19, while one extra step is included in the inventory but is not part of the main section of the inventory: the reactivation of the activated carbon used in the off-gas cleaning, which is the process by which the organics are recovered.

Table S19. Li-ion 18650 EOL process inventory.

Item		Flow	Unit	Reference process
**Functional unit**	**EOL**	1000	kg	
**Processes**	Discharging	2.9E-07	pc	
	Pyrometallurgical	1	pc	
	Mechanical separation combined unit	1	pc	
	Off-gas cleaning	1	pc	
**Infrastructure**	Facility	1.88E-08	p	*precious metal refinery construction/SE*

1.1.2.1. Discharging

To reach full discharge, the batteries are submerged in a solution of potassium hydroxide and water for two weeks. The solution, as stated by Accurec, can be reused an indefinite number of times.

Therefore, for linking a small share of the impact to each battery, it was assumed that the solution is used for eight years with an annual production capacity of 2 million metric tons [9].

Table S20. Li-ion discharging process inventory.

Item		Flow	Unit	Reference process
**Functional unit**	Discharging	1	pc	
**Materials**	KOH flakes	26	kg	*market for potassium hydroxide/GLO*
	Water	1024	kg	*water production and supply, decarbonised/RER*
**Energy & processes**	Electricity for discharging	1.67	kWh	*market for electricity, medium voltage/DE*

1.1.2.2. Pyrometallurgical process

After the complete discharge, the batteries undergo a pyrometallurgical process for 2 h. From this process, the organic components of the cell (i.e. the electrolyte and the separator) are trapped. Throughout the high-temperature thermal treatment, the only input required is electricity which is needed to reach the desired temperature allowing the full recovery of the organics.

Table S21. Li-ion pyrometallurgical process inventory.

Item		Flow	Unit	Reference process
**Functional unit**	Pyrometallurgical process	1	pc	
**Energy & processes**	Electricity for pyrometallurgy	269	kWh	*market for electricity, medium voltage/DE*

1.1.2.3. Off-gas cleaning

To recover the organic materials, an off-gas cleaning recovery process using activated carbon is applied. This step and the activated carbon, plus its recovery, are included in the inventory. However, the further final disposal of the organics trapped is outside of the system boundaries, thus not considered, meaning that neither burdens nor credits are given. The porous material needed for capturing the organics is dependent on the expected amount of materials that is supposed to trap. Indeed, for 1 kg that should be trapped, 1.6 kg of activated carbon is used. The activated carbon, once reactivated, can be reused for the same purpose. In the literature, no data is available regarding the number of times that the activated carbon can be reused with the same efficiency and for the same purpose. Therefore, it was assumed that the activated carbon can be reused 100 times without losing the needed characteristic. The activated carbon was modelled from scratch, using the ecoinvent centre [14] as a reference.

Table S22. Li-ion off-gas cleaning process inventory.

Item		Flow	Unit	Reference process
**Functional unit**	**Off-gas cleaning**	**1**	**pc**	
**Materials**	Crude coal	10.05	kg	*market for hard coal/AU*
	Water	41.54	kg	*water production and supply, decarbonised/RER*
**Energy & processes**	Electricity	1.84	kWh	*market for electricity, medium voltage/DE*
	Steam	44.56	MJ	*heat production, natural gas, at industrial furnace low-NOx >100kW /EU without Switzerland*
**Infrastructure**	Facility	3.21E-08	unit	*market for industrial furnace, coal, 1-10MW/GLO*
**Stressor**	Hard coal ash	−0.6	kg	*treatment of hard coal ash/CH*

1.1.2.4. Reactivation of spent activated carbon

The organic materials captured by the activated carbon cannot be recovered. However, the saturated activated carbon can be reactivate using water and electricity. Throughout the reactivation, ashes are generated due to the high temperature at which the spent activated carbon is exposed, and this ash was accounted for in the inventory. The amounts of ash, electricity and water used for the reactivation were gathered from the literature and compiled specifically for this inventory [14].

Table S23. Li-ion reactivation of spent activated carbon process inventory.

Item		Flow	Unit	Reference process
Functional unit	Reactivation of spent a.c.	469.57	kg	
**Materials**	Water	20.76	kg	*water production and supply, decarbonised/RER*
**Energy**	Electricity	2.82	kWh	*market for electricity, medium voltage/DE*
	Steam heating	22.88	MJ	*heat production, natural gas, at industrial furnace low-NOx >100kW/EU without Switzerland*
**Infrastructure**	Facility	1.77E-09	unit	*market for industrial furnace, coal, 1-10MW/GLO*
**Stressor**	Hard coal ash	0.04	kg	*treatment of hard coal ash/CH*

1.1.2.5. Mechanical separation combined unit

Following the pyrometallurgy, the materials left undergo five differentiated mechanical treatments that in the system modelled were assumed as a single-step process. From these steps, the cell container, made of steel, is recovered, as well as the electrode powder and both current collectors, made of Al and Cu. In all the steps, electricity and heat are needed, and since the EOL treatment occurs in Germany, the German electricity mix is assumed as a source of energy. As can be seen in Table S24, the values for the recovered materials were set to 0 to be consistent with the scenario here modelled, i.e., no benefits or burdens are given to the recycling process.

Table S24. Li-ion mechanical separation combined unit process inventory.

Item		Flow	Unit	Reference process
**Functional unit**	Mechanical separation combined unit	1	pc	
**Machinery**	Steel	1180	kg	*steel, low-alloyed/market for steel, low-alloyed/GLO*
**Energy & processes**	Electricity for mechanical processing	12	kWh	*market for electricity, medium voltage/DE*
**Outputs**	Electrode powder (fine fraction)	0	kg	*No process found in ecoinvent.*
	Steel (cell housing)	0	kg	*market for scrap steel/GLO*
	Al foils	0	kg	*market for waste aluminum/GLO*
	Cu foils	0	kg	*copper scrap, sorted, pressed/GLO*

Finally, the data provided by our partner Accurec was designed for a battery cell with slightly different material loadings compared with the reference cell we used for the assessment of the production and use phase. To maintain consistency, we adjusted the recycling process, both in terms of energy used and materials recovered, to simulate the recycling process of the reference cell. The different material loading mainly affected the mechanical process, due to a higher demand of

electricity and to the off-gas cleaning phase, whereas higher amounts of energy and activated carbon are needed to trap effectively the surplus of organic material.

2. Complete Numerical Results

2.1. Battery production phase

Table S24 presents the numerical results from the environmental characterization of the two assessed chemistries for the following eight impact categories: global warming potential (GWP), metal depletion potential (MDP), human toxicity potential (HTP), fossil depletion potential (FDP), freshwater eutrophication potential (FEP), marine eutrophication potential (MEP), marine toxicity potential (METP) and freshwater toxicity potential (FETP). Figure S7 illustrates the normalized relative contribution of the five components plus the battery manufacture process and compares simultaneously the impact of both chemistries.

Table S25. Production impact of Al-ion and Li-ion cells.

Impact	Chemistry	Electrolyte	Anode	Cathode	Housing	Separator	Assembly
GWP	Al-ion	3,74E-01	9,83E-02	3,91E-02	2,20E-01	6,08E-02	1,83E+00
[Kg CO2-eq]	Li-ion	3,68E-03	1,23E-02	3,25E-02	1,31E-02	6,12E-04	1,83E-01
MDP	Al-ion	1,09E-02	2,34E-03	1,27E-03	4,53E-01	2,87E-03	1,65E-02
[Kg Fe-eq]	Li-ion	3,44E-04	7,31E-02	7,44E-02	2,87E-02	2,85E-05	1,69E-03
HTP	Al-ion	1,29E-01	4,78E-02	2,07E-02	1,09E-01	1,64E-02	7,80E-01
[Kg 1.4 DB-eq]	Li-ion	2,83E-03	6,32E-01	4,37E-02	5,94E-03	1,74E-04	8,52E-02
FDP	Al-ion	8,46E-02	2,19E-02	1,88E-02	5,30E-02	3,08E-02	4,22E-01
[Kg oil-eq]	Li-ion	1,36E-03	3,46E-03	7,44E-03	3,16E-03	4,44E-04	4,74E-02
FEP	Al-ion	1,41E-04	6,02E-05	2,83E-05	9,54E-05	1,29E-05	1,12E-03
[Kg P-eq]	Li-ion	1,81E-06	2,82E-04	3,53E-05	5,24E-06	1,41E-07	1,24E-04
MEP	Al-ion	6,40E-05	2,44E-05	9,90E-06	5,94E-05	1,66E-04	4,45E-04
[Kg N-eq]	Li-ion	1,37E-06	3,04E-05	1,61E-05	3,43E-06	8,33E-08	4,84E-05
METP	Al-ion	5,83E-03	2,38E-03	6,17E-04	1,47E-02	5,44E-04	1,90E-02
[Kg 1.4 DB-eq]	Li-ion	8,51E-05	1,01E-02	3,09E-05	9,17E-04	4,97E-06	2,60E-03
FETP	Al-ion	6,75E-03	2,55E-03	6,54E-04	1,48E-02	5,82E-04	2,01E-02
[Kg 1.4 DB-eq]	Li-ion	7,81E-05	9,52E-03	1,74E-03	9,19E-04	5,27E-06	2,81E-03

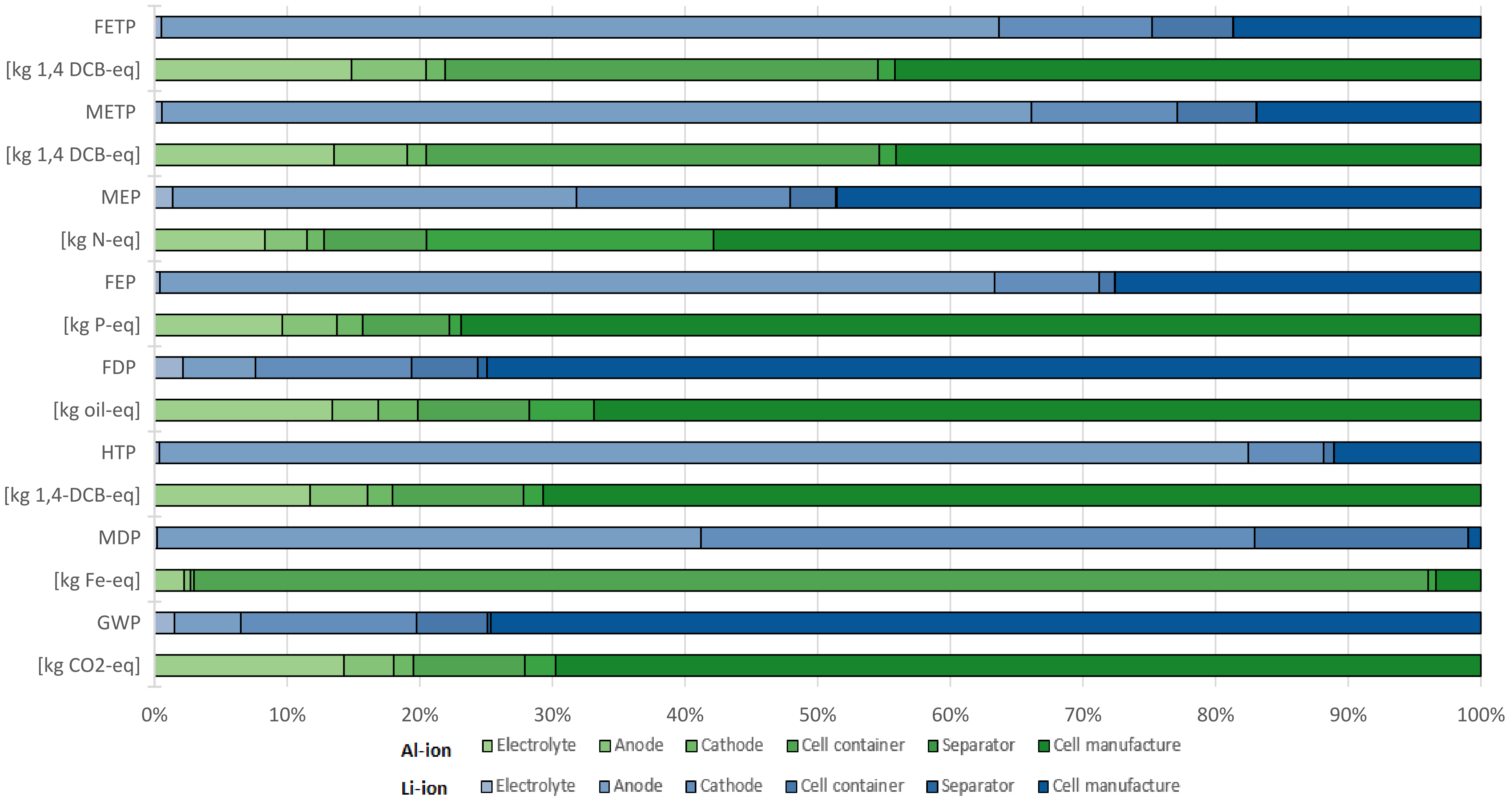

Figure S7. Production phase contribution analysis for eight impact categories of Li-ion and Al-ion 18650 cells.

2.2. Battery recycling

Following Table S26 presents the numerical results regarding the end-of-life phase for the following impact categories: global warming potential (GWP), metal depletion potential (MDP), human toxicity potential (HTP), fossil depletion potential (FDP), freshwater eutrophication potential (FEP), marine eutrophication potential (MEP), marine toxicity potential (METP) and freshwater toxicity potential (FETP). Figure S8 illustrates the normalized relative contribution of the five stages involve in the Al-ion cells recycling process.

Table S26. Environmental impacts due to the recycling process of Al-ion batteries.

**Impact**	**Discharging**	**VS + LHT**	**MSCU**	**Off-gas cleaning**	
				Activated carbon	Reactivation of spent activated carbon
GWP	5,83E+01	1,21E+02	5,07E+00	2,68E+01	1,53E+02
MDP	2,96E+00	1,20E+00	5,00E-02	2,39E-01	1,33E+00
HTP	2,68E+01	9,23E+01	3,86E+00	2,21E+01	6,70E+01
FDP	1,56E+01	3,03E+01	1,27E+00	9,55E+00	4,72E+01
FEP	3,12E-02	1,53E-01	6,39E-03	2,70E-02	1,01E-01
MEP	1,19E-02	3,82E-02	1,60E-03	6,83E-03	2,72E-02
METP	9,63E-01	2,76E+00	1,15E-01	5,95E-01	2,05E+00
FETP	1,04E+00	2,95E+00	1,23E-01	6,37E-01	2,19E+00

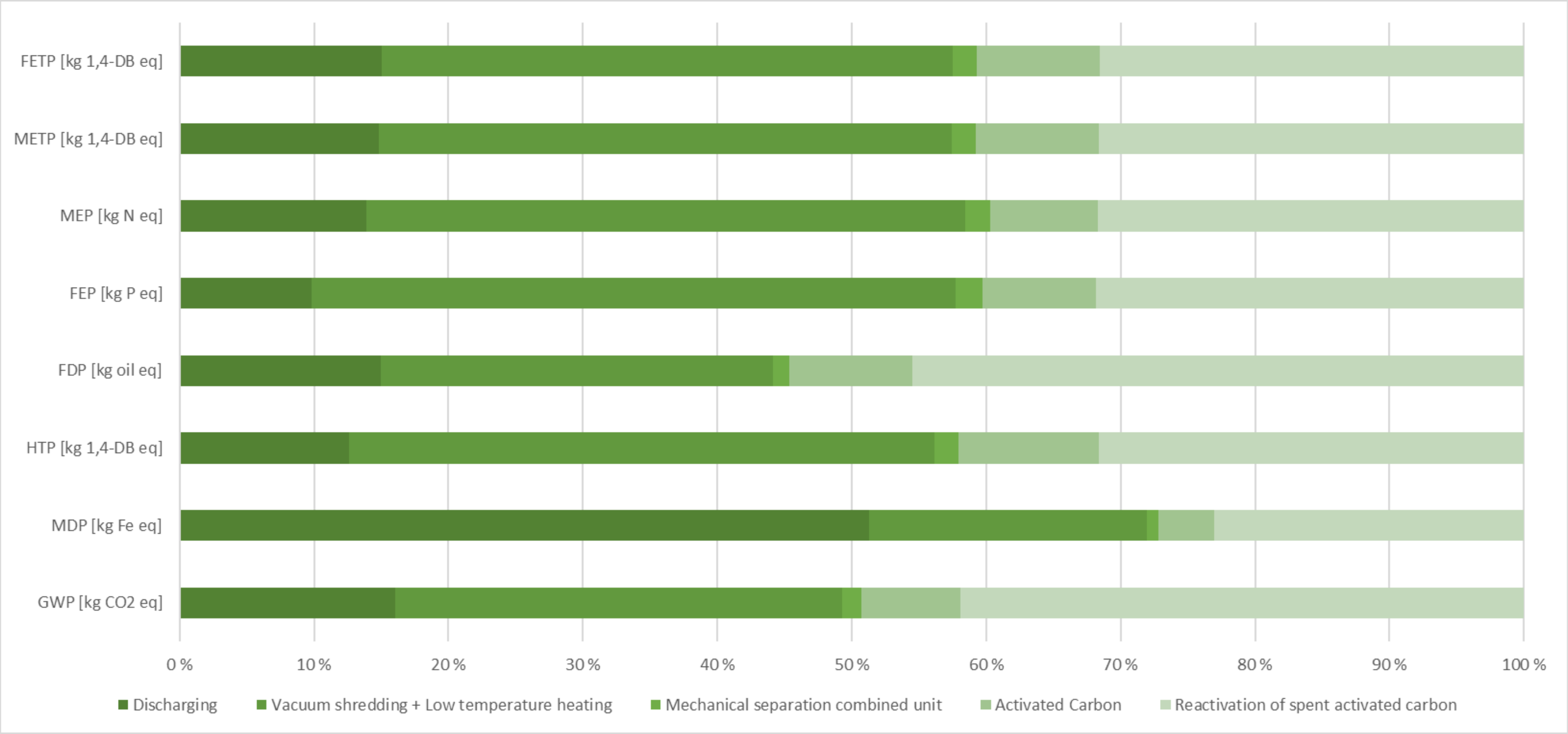

Figure S8. Contribution analysis for eight impact categories of the Al-ion recycling process.

2.3. *Sensitivity analysis*

Table S27 displays the numerical results from the sensitivity analysis for a GWP indicator. In the analysis we simulated a scenario in which both Al-ion and Li-ion cells are manufactured under the same conditions as in a Tesla Gigafactory (clean production scenario)—that is, using 100% PV energy. Thus, the analysis is carried out comparing the results from the LCA manufacturing phase (baseline scenario) and the clean production scenario.

Table S27. Sensitivity analysis.

	Functional unit	Scenario	Electrolyte [Kg CO_2-eq]	Anode [Kg CO_2-eq]	Cathode [Kg CO_2-eq]	Housing [Kg CO_2-eq]	Separator [Kg CO_2-eq]	Assembly [Kg CO_2-eq]
Al-ion	Per Wh	Baseline	3,74E-01	9,83E-02	3,91E-02	2,20E-01	6,08E-02	1,83E+00
	Per Wh	Clean P.	3,74E-01	9,83E-02	3,91E-02	2,20E-01	6,08E-02	1,60E-01
	Per Cell	Baseline	1,01E-01	2,65E-02	1,06E-02	5,94E-02	1,64E-02	4,94E-01
	Per Cell	Clean P.	1,01E-01	2,65E-02	1,06E-02	5,94E-02	1,64E-02	4,32E-02
Li-ion	Per Wh	Baseline	3,68E-03	1,23E-02	3,25E-02	1,31E-02	6,12E-04	1,83E-01
	Per Wh	Clean P.	3,68E-03	1,23E-02	3,25E-02	1,31E-02	6,12E-04	1,82E-02
	Per Cell	Baseline	1,99E-02	6,62E-02	1,75E-01	7,08E-02	3,31E-03	9,88E-01
	Per Cell	Clean P.	1,99E-02	6,62E-02	1,75E-01	7,08E-02	3,31E-03	9,85E-02

2.4. *Uncertainty analysis*

Table S28 shows the numerical results of the uncertainty analysis. The scenarios were modelled considering the following assumptions: first, the baseline scenario is assumed as the current energy intensity of the Li-ion cell. Second, a 30% reduction in energy usage is considered for the Al-ion cell due to the absence of a dry room in the manufacturing process. Finally, energy usage is assumed to exhibit a substantial reduction, to 10 kWh/kg. Hence, with the three different energy intensities assumed, the GHG impacts are framed within a reasonable spectrum.

Table S28. Sensitivity analysis.

	Functional unit	Scenario	Materials [Kg CO2 eq]	Energy [Kg CO2 eq]
Al-ion	Per Wh	10 kWh/Kg	7,05E-01	7,81E-01
	Per Wh	24 kWh/Kg	7,05E-01	1,83E+00
	Per Wh	34 kWh/Kg	7,05E-01	2,51E+00
	Per Cell	10 kWh/Kg	1,90E-01	2,11E-01
	Per Cell	24 kWh/Kg	1,90E-01	4,94E-01
	Per Cell	34 kWh/Kg	1,90E-01	6,79E-01
Li-ion	Per Wh	10 kWh/Kg	6,21E-02	5,52E-02
	Per Wh	24 kWh/Kg	6,21E-02	1,32E-01
	Per Wh	34 kWh/Kg	6,21E-02	1,83E-01
	Per Cell	10 kWh/Kg	3,36E-01	2,98E-01
	Per Cell	24 kWh/Kg	3,36E-01	7,12E-01
	Per Cell	34 kWh/Kg	3,36E-01	9,88E-01

References

1. Ellingsen L A-W, Holland A, Drillet J-F, Peters W, Eckert M, Concepcion C, Ruiz O, Colin J-F, Knipping E, Pan Q, Wills R and Majeau-Bettez G 2018 Environmental Screening of Electrode Materials for a Rechargeable Aluminum Battery with an AlCl3/EMIMCl Electrolyte *Materials (Basel).* **11** 936
2. Johnson, M.C.; Sullivan J L 2017 Lightweight Materials for Auotomotive Application *Argonne Natl. Lab.* **91** 399–404
3. Laird T 1997 Ullmann's Encyclopedia of Industrial Chemistry, 5th Edition VCH: Weinheim, Germany. 1996/1997. Section A, 28 vols. Section B, 8 vols. DM 19 400. *Org. Process Res. Dev.* **1** 391–2
4. Solvionic, Toulouse, France, Personal Communication, 2018
5. Werner F, Umwelt W, Werner F and Umwelt W 2015 Ecoinvent 3.5 dataset documentation *Ecoinvent Cent.* 1–7
6. Reuss G, Disteldorf W, Gamer A O and Hilt A 2012 Fluorine Compounds, Organic *Ulmann's Encycl. iIndustrial Chem.*
7. Ebel K, Koehler H, Gamer A O and Jäckh R 2000 Imidazole and Derivatives *Ullmann's Encyclopedia of Industrial Chemistry*
8. Howells R D and Mc Cown J D 1977 Trifluoromethanesulfonic Acid and Derivatives *Chem. Rev.*
9. Accurec, Heißen, Germany; Personal Communication, 2018
10. Steubing B, Wernet G, Reinhard J, Bauer C and Moreno-Ruiz E 2016 The ecoinvent database version 3 (part II): analyzing LCA results and comparison to version 2 *Int. J. Life Cycle Assess.* **21** 1269–81
11. Ellingsen L A-W, Majeau-Bettez G, Singh B, Srivastava A K, Valøen L O and Strømman A H 2014 Life cycle assessment of a lithium-ion battery vehicle pack *J. Ind. Ecol.* **18** 113–124
12. Ellingsen L A W, Majeau-Bettez G, Singh B, Srivastava A K, Valøen L O and Strømman A H 2014 Life Cycle Assessment of a Lithium-Ion Battery Vehicle Pack *J. Ind. Ecol.* **18** 113–24
13. G. Energy; Nesttund, Norway, Personal Communication, 2016
14. Treyer K 2016 Ecoinvent 3.3 dataset documentation **i** 2015–6

© 2019 by the authors. Submitted for possible open access publication under the terms and conditions of the Creative Commons Attribution (CC BY) license (http://creativecommons.org/licenses/by/4.0/).
